# Skin Reinnervation by Collateral Sprouting Following Spared Nerve Injury in Mice

**DOI:** 10.1523/JNEUROSCI.1494-23.2024

**Published:** 2024-03-12

**Authors:** Sang-Min Jeon, Aishwarya Pradeep, Dennis Chang, Leah McDonough, Yijia Chen, Alban Latremoliere, LaTasha K. Crawford, Michael J. Caterina

**Affiliations:** ^1^Department of Neurosurgery, Neurosurgery Pain Research Institute, Johns Hopkins School of Medicine, Baltimore, Maryland 21205; ^2^Department of Neuroscience, Johns Hopkins School of Medicine, Baltimore, Maryland 21205; ^3^Department of Pathological Sciences, University of Wisconsin-Madison School of Veterinary Medicine, Madison, Wisconsin 53706; ^4^Department of Biological Chemistry, Johns Hopkins School of Medicine, Baltimore, Maryland 21205

**Keywords:** collateral sprouting, innervation, mechanoreceptor, nociceptive, peptidergic, peripheral nerve injury

## Abstract

Following peripheral nerve injury, denervated tissues can be reinnervated via regeneration of injured neurons or collateral sprouting of neighboring uninjured afferents into denervated territory. While there has been substantial focus on mechanisms underlying regeneration, collateral sprouting has received less attention. Here, we used immunohistochemistry and genetic neuronal labeling to define the subtype specificity of sprouting-mediated reinnervation of plantar hindpaw skin in the mouse spared nerve injury (SNI) model, in which productive regeneration cannot occur. Following initial loss of cutaneous afferents in the tibial nerve territory, we observed progressive centripetal reinnervation by multiple subtypes of neighboring uninjured fibers into denervated glabrous and hairy plantar skin of male mice. In addition to dermal reinnervation, CGRP-expressing peptidergic fibers slowly but continuously repopulated denervated epidermis, Interestingly, GFRα2-expressing nonpeptidergic fibers exhibited a transient burst of epidermal reinnervation, followed by a trend towards regression. Presumptive sympathetic nerve fibers also sprouted into denervated territory, as did a population of myelinated TrkC lineage fibers, though the latter did so inefficiently. Conversely, rapidly adapting Aβ fiber and C fiber low threshold mechanoreceptor (LTMR) subtypes failed to exhibit convincing sprouting up to 8 weeks after nerve injury in males or females. Optogenetics and behavioral assays in male mice further demonstrated the functionality of collaterally sprouted fibers in hairy plantar skin with restoration of punctate mechanosensation without hypersensitivity. Our findings advance understanding of differential collateral sprouting among sensory neuron subpopulations and may guide strategies to promote the progression of sensory recovery or limit maladaptive sensory phenomena after peripheral nerve injury.

## Significance Statement

Following nerve injury, whereas one mechanism for tissue reinnervation is regeneration of injured neurons, another, less well studied mechanism is collateral sprouting of nearby uninjured neurons. In this study, we examined collateral sprouting in denervated mouse skin and showed that it involves some, but not all neuronal subtypes. Despite such heterogeneity, a significant degree of restoration of punctate mechanical sensitivity is achieved. These findings highlight the diversity of collateral sprouting among peripheral neuron subtypes and reveal important differences between pre- and postdenervation skin that might be appealing targets for therapeutic correction to enhance functional recovery from denervation and prevent unwanted sensory phenomena such as pain or numbness.

## Introduction

Injury to peripheral nerves can not only impair motor function but also interfere with sensory functions necessary for protective reflexes and tactile acuity. Furthermore, nerve injury can lead to abnormal neuronal excitability and consequent neuropathic pain (Ji and Strichartz, 2004; Campbell and Meyer, 2006; Costigan et al., 2009). Recovery from nerve injury occurs via two distinct processes. In the first of these, regeneration, injured axons grow along their original paths to reinnervate target tissues (Rigoni and Negro, 2020). While this represents one potential means of achieving restoration of sensory function, it is sometimes impossible, due to an excessive gap in the injured nerve. Even when regeneration is possible, if a nerve is injured far from its target tissues, the inherently slow rate of regeneration delays and might even preclude functional restoration (Nath and Mackinnon, 2000). A second mechanism of reinnervation, collateral sprouting, involves local ectopic branching and axonal extension from uninjured (i.e., spared) neurons into adjacent denervated tissue (Livingston, 1947; Ahcan et al., 1998; Theriault et al., 1998; Ali et al., 2002). Because it is initiated locally, collateral sprouting theoretically offers the opportunity for a more rapid sensory recovery than nerve regeneration, and could compensate when regeneration is impossible or delayed (Ahcan et al., 1998). However, available data suggest that collateral sprouting in humans is limited in extent, and that in both humans and in animal models, collateral sprouting is nonuniform across sensory modalities and neuronal subtypes (Horch, 1981; Jackson and Diamond, 1984; Diamond and Foerster, 1992; Ahcan et al., 1998). There is also a complex relationship between tissue reinnervation and the duration of neuropathic pain, and in some cases neuropathic pain may occur in regions of collateral sprouting (Ali et al., 2002; Gangadharan et al., 2022). Given these considerations, elucidation of mechanisms governing both regeneration and collateral sprouting is necessary to facilitate the restoration of desirable sensory function and may help avoid or reverse the development of postinjury pathological pain.

In this study, we used whole-mount tissue staining, genetically labeled mouse lines, and both conventional and optogenetic behavioral assays to anatomically and functionally assess collateral sprouting in the mouse spared nerve injury model. Following initial denervation, we observed significant centripetal sprouting by some, but not all subpopulations of sensory neurons assayed. Moreover, while peptidergic nociceptors exhibited a slow, steady timecourse of epidermal reinnervation, nonpeptidergic fibers showed a transient burst of such reinnervation that subsequently regressed. Meanwhile, we observed a paucity or lack of sprouting among some low-threshold mechanoreceptor subtypes. Despite such heterogeneity, we observed apparently faithful restoration of punctate mechanosensitivity across a wide range of forces in this skin territory without evident overshoot. Together, our data highlight the subtype specificity of collateral sprouting and illustrate the sufficiency of those fibers that do sprout to restore significant sensitivity to denervated hindpaw skin.

## Materials and Methods

### Mouse strains

TH^2ACreER^, Split^Cre^, TrkC^tdTomato^, TrkC^CreER^ mouse lines (Li et al., 2011; Rutlin et al., 2014; Bai et al., 2015; Abraira et al., 2017) were generously provided by Dr. David Ginty. Pirt^Cre^ mice (Kim et al., 2014) were kindly provided by Dr. Xinzhong Dong. Rosa26^LSLEYFP^ (Ai3, #007903), Rosa26^LSLtdTomato^ (Ai9, #007909), Rosa26^LSL-ChR2-EYFP^ (Ai32, #024109) and C57BL/6J (#000664) mice were obtained from the Jackson Laboratory. Separate groups of male mice at least 7–8 weeks old at the start of the experiments, were used for behavioral and anatomical studies, respectively. In experiments using LTMR transgenic mice, both male and female mice were used. Age-matched mice from the same or parallel mating cages were randomly assigned to experimental groups in injured versus sham comparisons. Mice were housed 1–5 per cage on a 14 h light/10 h dark/light cycle, and provided food and water *ad libitum*. Mice were handled in accordance with the Johns Hopkins University Institutional Animal Care and Use Committee guidelines as well as National Institutes of Health Guide for the Care and Use of Laboratory Animals.

### Peripheral nerve injury model

Spared nerve injury (SNI) was performed as previously described (Bourquin et al., 2006). In brief, under deep isoflurane anesthesia, one sciatic nerve of mice 7–8 weeks old was exposed in the thigh region, near its trifurcation, and the tibial and common peroneal nerve branches were ligated. A small section immediately distal to the ligation was excised. The sural nerve branch was left intact, avoiding contact or stretching. Finally, muscle and skin were sutured in two distinct layers with silk 6-0 and 4-0 sutures, respectively. In a limited number of mice, a second surgical exposure of the sciatic nerve was performed 8 weeks after sural sparing SNI, and the remaining sural branch was transected; a small piece distal to the transection was excised, and muscle and skin were again closed in two layers. For sciatic nerve transection (SNT), the sciatic nerve was transected proximal to the separation of the three branches (common peroneal, tibial, and sural) from one another, and muscle and skin were closed in two layers.

### Behavioral testing

Experiments were performed at baseline (usually the day before surgery) and up to 2 months after SNI surgery. For assays using pinprick, 1.4 g von Frey filament, and optogenetic stimulation, experiments were performed at baseline and at intervals of 3 or 4 d for 1 month after SNI. For assays with von Frey filaments over the full range of forces, experiments were performed at baseline and at 1, 4, and 8 weeks after SNI. For assays using the 0.4 g von Frey filament, experiments were performed at baseline and weekly for 8 weeks after SNI. Behavioral assays for optogenetics were conducted with the experimenter blinded to genotype or to sham versus SNI treatment. Animal numbers for each experiment are indicated in the figures and in Table 1.

**Table 1.** Statistical analysis and the number of animals/samples used in the experiments

Figure	Pre hoc	Post hoc	*N* (number of samples/animals per group)
1*E*	(1) One-way ANOVA: *F*_(3, 13)_ = 7.374, *p* = 0.0039** (2) Two-way ANOVA: Time × Contra/Ipsi: *F*_(3, 13)_ = 3.205, *p* = 0.0588 Time: *F*_(3, 13)_ = 3.529, *p* = 0.0456* Contra/Ipsi: *F*_(1, 13)_ = 49.65, *p* < 0.0001****	Bonferroni's multiple-comparisons test 0 vs 1 week: *p* = 0.0031^++^ 0 vs 4 weeks: *p* = 0.0587 0 vs 8 week: *p* = 0.3175 Bonferroni's multiple-comparisons test 0, contra vs ipsi: *p* > 0.9999 1 week, contra vs ipsi: *p* = 0.0004*** 4 weeks, contra vs ipsi: *p* = 0.0072** 8 weeks, contra vs ipsi: *p* = 0.0137*	Naïve: 4 1 week: 4 4 weeks: 4 8 weeks: 5
1*F*	(1) One-way ANOVA: *F*_(3, 11)_ = 5.726, *p* = 0.0131* (2) Two-way ANOVA: Time × Contra/Ipsi: *F*_(3, 11)_ = 3.505, *p* = 0.0530 Time: *F*_(3, 11)_ = 1.124, *p* = 0.3814 Contra/Ipsi: *F*_(1, 11)_ = 12.83, *p* = 0.0043**	Bonferroni's multiple-comparisons test 0 vs 1 week: *p* = 0.0365^+^ 0 vs 4 weeks: >0.9999 0 vs 8 weeks: >0.9999 Bonferroni's multiple-comparisons test 0, contra vs ipsi: *p* > 0.9999 1 week, contra vs ipsi: *p* = 0.0028** 4 weeks, contra vs ipsi: *p* = 0.5453 8 weeks, contra vs ipsi: *p* > 0.9999	Naïve: 4 1 week: 4 4 weeks: 4 8 weeks: 3
1*G*	(1) One-way ANOVA: *F*_(3, 13)_ = 2.927, *p* = 0.0736 (2) Two-way ANOVA: Time × Contra/Ipsi: *F*_(3, 13)_ = 2.092, *p* = 0.1507 Time: *F*_(3, 13)_ = 2.190, *p* = 0.1381 Contra/Ipsi: *F*_(1, 13)_ = 18.28, *p* = 0.0009***	Bonferroni's multiple-comparisons test 0, contra vs ipsi: *p* > 0.9999 1 week, contra vs ipsi: *p* = 0.1655 4 weeks, contra vs ipsi: 0.0288* 8 weeks, contra vs ipsi: 0.0273*	Naïve: 4 1 week: 4 4 weeks: 4 8 weeks: 5
1*H*	(1) One-way ANOVA: *F*_(3, 11)_ = 0.6940, *p* = 0.5747 (2) Two-way ANOVA: Time × Contra/Ipsi: *F*_(3, 11)_ = 0.3097, *p* = 0.8180 Time: *F*_(3, 11)_ = 1.513, *p* = 0.2656 Contra/Ipsi: *F*_(1, 11)_ = 1.462, *p* = 0.2520		Naïve: 4 1 week: 4 4 weeks: 4 8 weeks: 3
1*I*	(1) One-way ANOVA: *F*_(3, 13)_ = 43.05, *p* < 0.0001**** (2) Two-way ANOVA: Time × Contra/Ipsi: *F*_(3, 13)_ = 28.13, *p* = 0.0002 Time: *F*_(3, 13)_ = 28.13, *p* < 0.0001 Contra/Ipsi: *F*_(1, 13)_ = 69.05, *p* < 0.0001****	Bonferroni's multiple-comparisons test 0 vs 1 week: *p* < 0.0001^++++^ 0 vs 4 weeks: *p* < 0.0001^++++^ 0 vs 8 weeks: *p* = 0.0141^+^ Bonferroni's multiple-comparisons test 0, contra vs ipsi: *p* > 0.9999 1 week, contra vs ipsi: *p* < 0.0001**** 4 weeks, contra vs ipsi: *p* < 0.0001**** 8 weeks, contra vs ipsi: *p* = 0.0758	Naïve: 4 1 week: 4 4 weeks: 4 8 weeks: 5
1*J*	(1) One-way ANOVA: *F*_(3, 11)_ = 22.69, *p* < 0.0001**** (2) Two-way ANOVA: Time × Contra/Ipsi: *F*_(3, 11)_ = 15.12, *p* = 0.0003*** Time: *F*_(3, 11)_ = 11.67, *p* = 0.0010*** Contra/Ipsi: *F*_(1, 11)_ = 40.71, *p* < 0.0001****	Bonferroni's multiple-comparisons test 0 vs 1 week: *p* < 0.0001^++++^ 0 vs 4 weeks: *p* = 0.0052^++^ 0 vs 8 weeks: *p* > 0.9999 Bonferroni's multiple-comparisons test 0, contra vs ipsi: *p* > 0.9999 1 week, contra vs ipsi: *p* = 0.0002*** 4 weeks, contra vs ipsi: *p* < 0.0001**** 8 weeks, contra vs ipsi: *p* > 0.9999	Naïve: 4 1 week: 4 4 weeks: 4 8 weeks: 3
2*C*	Two-way ANOVA: Type of CGRP × contra/Ipsi: *F*_(1, 8)_ = 8.614, *p* = 0.0189 Type of CGRP: *F*_(1, 8)_ = 231.5, *p* = 0.0001**** Contra ipsi: *F*_(1, 8)_ = 0.000, *p* > 0.9999		C57Bl6: 5
2*D*	Two-way ANOVA: Type of NFH × contra/Ipsi: *F*_(1, 8)_ = 1.698, *p* = 0.2288 Type of NFH: *F*_(1, 8)_ = 6.205, *p* = 0.0375* Contra ipsi: *F*_(1, 8)_ = 0.000, *p* > 0.9999		C57Bl6: 5
2*E*	Pearson correlation: *r* = 0.06263%, 95% confidence interval = −0.8676 to 0.8954, R squared = 0.003923, P (two-tailed) = 0.9203		Total NFH: 5 NFH^+^CGRP^+^: 5
2*F*	Pearson correlation: *r* = 0.1041%, 95% confidence interval = −0.8569 to 0.9034, R squared = 0.01083, P (two-tailed) = 0.8677		Total NFH: 5 NFH^+^CGRP^+^: 5
2*H*	(1) One-way ANOVA: *F*_(3, 12)_ = 6.640, *p* < 0.0068** (2) Two-way ANOVA: Time × Contra/Ipsi: *F*_(3, 12)_ = 10.52, *p* = 0.0011** Time: *F*_(3, 12)_ = 2.374, *p* = 0.1214 Contra/Ipsi: *F*_(1, 12)_ = 43.81, *p* < 0.0001****	Bonferroni's multiple-comparisons test 0 vs 1 week: *p* < 0.0054^++^ 0 vs 4 weeks: *p* = 0.0854 0 vs 8 weeks: *p* = 0.1098 Bonferroni's multiple-comparisons test 0, contra vs ipsi: *p* > 0.9999 1 week, contra vs ipsi: *p* = 0.0001*** 4 weeks, contra vs ipsi: *p* = 0.0024** 8 weeks, contra vs ipsi: *p* = 0.0173*	Naïve: 4 1 week: 4 4 weeks: 4 8 weeks: 4
2*I*	(1) One-way ANOVA: *F*_(3, 12)_ = 1.190, *p* = 0.3550 (2) Two-way ANOVA: Time × Contra/Ipsi: *F*_(3, 12)_ = 0.4912, *p* = 0.6950 Time: *F*_(3, 12)_ = 2.490, *p* = 0.1101 Contra/Ipsi: *F*_(1, 12)_ = 1.258, *p* = 0.2839		Naïve: 4 1 week: 4 4 weeks: 4 8 weeks: 4
2*J*	(1) One-way ANOVA: *F*_(3, 12)_ = 19.25, *p* < 0.0001**** (2) Two-way ANOVA: Time × Contra/Ipsi: *F*_(3, 12)_ = 14.90, *p* = 0.0002*** Time: *F*_(3, 12)_ = 6.334, *p* = 0.0081** Contra/Ipsi: *F*_(1, 12)_ = 71.04, *p* < 0.0001****	Bonferroni's multiple-comparisons test 0 vs 1 week: *p* < 0.0001^+++^ 0 vs 4 weeks: *p* = 0.0002^+++^ 0 vs 8 weeks: *p* = 0.0086^++^ Bonferroni's multiple-comparisons test 0, contra vs ipsi: *p* > 0.9999 1 week, contra vs ipsi: *p* < 0.0001**** 4 weeks, contra vs ipsi: *p* < 0.0001**** 8 weeks, contra vs ipsi: *p* = 0.5010	Naïve: 4 1 week: 4 4 weeks: 4 8 weeks: 4
4*C*	(1) Unpaired two-tailed *t* test 1 W ipsi vs 8 W ipsi: *t* = 5.656, df = 6, *p* = 0.0013** (2) Two-way ANOVA: Time × Contra/Ipsi: *F*_(1, 6)_ = 6.825, *p* = 0.0400* Time: *F*_(1, 6)_ = 6.148, *p* = 0.0478* Contra/Ipsi: *F*_(1, 6)_ = 75.25, *p* = 0.0001***	Bonferroni's multiple-comparisons test 1 week, contra vs ipsi: *p* = 0.0004*** 8 weeks, contra vs ipsi: *p* = 0.0103*	1 week: 4 8 weeks: 4
4*D*	Paired two-tailed *t* test CGRP: *t* = 3.838, df = 3, *p* = 0.0312* NFH: *t* = 3.910, df = 3, *p* = 0.0297* IB4: *t* = 7.353, df = 3, *p* = 0.0052** NFH-CGRP+: *t* = 3.003, df = 3, *p* = 0.0575		8 weeks: 4
4*E*	Paired two-tailed *t* test CGRP: *t* = 2.250, df = 3, *p* = 0.1100 NFH: *t* = 0.6351, df = 3, *p* = 0.6351 IB4: *t* = 2.177, df = 3, *p* = 0.1176 NFH-CGRP+: *t* = 0.2720, df = 3, *p* = 0.8033		8 weeks: 4
5*F*	One-way ANOVA: *F*_(3, 12)_ = 84.51, *p* < 0.0001***	Bonferroni's multiple-comparisons test 0 vs 1 week: *p* < 0.0001**** 0 vs 4 weeks: *p* < 0.0001**** 0 vs 8 weeks: *p* < 0.0001**** 1 vs 4 weeks: *p* = 0.9084 1 vs 8 weeks: *p* = 0.0302^+^ 4 vs 8 weeks: *p* = 0.4965	Naïve: 4 1 week: 4 4 weeks: 4 8 weeks: 4
5*G*	One-way ANOVA: *F*_(3, 12)_ = 15.85, *p* = 0.0002***	Bonferroni's multiple-comparisons test 0 vs 1 week: *p* < 0.0003*** 0 vs 4 weeks: *p* = 0.2921 0 vs 8 weeks: *p* = 0.0017** 1 vs 4 weeks: *p* = 0.0098^++^ 1 vs 8 weeks: *p* > 0.9999 4 vs 8 weeks: *p* = 0.0867	Naïve: 4 1 week: 4 4 weeks: 4 8 weeks: 4
6*D*	(1) One-way ANOVA: *F*_(3, 20)_ = 11.78, *p* = 0.0001*** (2) Two-way ANOVA: Time × Contra/Ipsi: *F*_(3, 20)_ = 2.789, *p* = 0.0671 Time: *F*_(3, 20)_ = 5.211, *p* = 0.0080** Contra/Ipsi: *F*_(1, 20)_ = 24.13, *p* < 0.0001****	Bonferroni's multiple-comparisons test 0 vs 1 week: *p* = 0.0011^++^ 0 vs 4 weeks: *p* = 0.0013^++^ 0 vs 8 weeks: *p* = 0.0002^+++^ Bonferroni's multiple-comparisons test 0, contra vs ipsi: *p* > 0.9999 1 week, contra vs ipsi: *p* = 0.0009*** 4 weeks, contra vs ipsi: *p* = 0.1591 8 weeks, contra vs ipsi: *p* = 0.1378	Naïve: 6 1 week: 5 4 weeks: 6 8 weeks: 7
6*E*	(1) One-way ANOVA: *F*_(3, 20)_ = 18.62, *p* < 0.0001**** (2) Two-way ANOVA: Time × Contra/Ipsi: *F*_(3, 20)_ = 3.545, *p* = 0.0331* Time: *F*_(3, 20)_ = 6.848, *p* = 0.0023** Contra/Ipsi: *F*_(1, 20)_ = 57.84, *p* < 0.0001****	Bonferroni's multiple-comparisons test 0 vs 1 week: *p* < 0.0001^++++^ 0 vs 4 weeks: *p* < 0.0001^++++^ 0 vs 8 weeks: *p* < 0.0001^++++^ Bonferroni's multiple-comparisons test 0, contra vs ipsi: *p* > 0.9999 1 week, contra vs ipsi: *p* = 0.0002*** 4 weeks, contra vs ipsi: *p* = 0.0208* 8 weeks, contra vs ipsi: *p* = 0.0001***	Naïve: 6 1 week: 5 4 weeks: 6 8 weeks: 7
6*I*	(1) One-way ANOVA: *F*_(3, 20)_ = 9.081, *p* = 0.0005**** (2) Two-way ANOVA: Time × Contra/Ipsi: *F*_(3, 20)_ = 11.92, *p* = 0.0001*** Time: *F*_(3, 20)_ = 2.407, *p* = 0.0973 Contra/Ipsi: *F*_(1, 20)_ = 77.85, *p* < 0.0001****	Bonferroni's multiple-comparisons test 0 vs 1 week: *p* = 0.0020^++^ 0 vs 4 weeks: *p* = 0.0012^++^ 0 vs 8 weeks: *p* = 0.0193^+^ Bonferroni's multiple-comparisons test 0, contra vs ipsi: *p* > 0.9999 1 week, contra vs ipsi: *p* = 0.0026** 4 weeks, contra vs ipsi: *p* < 0.0001**** 8 weeks, contra vs ipsi: *p* < 0.0001****	Naïve: 6 1 week: 4 4 weeks: 6 8 weeks: 8
6*L*	Two-way ANOVA: Type of NFH × contra/Ipsi: *F*_(1, 8)_ = 8.352, *p* = 0.0202 Type of NFH: *F*_(1, 8)_ = 10.96, *p* = 0.0107* Contra ipsi: *F*_(1, 8)_ = 1.289 × 10^−19^, *p* > 0.9999		TrkC^tdtomato^: 5
6*M*	Two-way ANOVA: Type of TrkC × contra/Ipsi: *F*_(1, 8)_ = 0.001700, *p* = 0.9681 Type of TrkC: *F*_(1, 8)_ = 21.39, *p* = 0.0017** Contra ipsi: *F*_(1, 8)_ = 2.290 × 10^−20^, *p* > 0.9999		TrkC^tdtomato^: 5
6*N*	Pearson correlation: *r* = −0.3809%, 95% confidence interval = −0.9454 to 0.7552, R squared = 0.1451, P (two-tailed) = 0.5271		Total TrkC: 5 TrkC^+^NFH^+^: 5
6*O*	Pearson correlation: *r* = 0.9861%, 95% confidence interval = 0.7985 to 0.9991, R squared = 0.9724, P (two-tailed) = 0.0020**		Total TrkC: 5 TrkC^+^NFH^+^: 5
7*H*	Paired *t* test: Saline vs 6-OHDA: *t* = 7.199, df = 4, *p* = 0.0020**		Saline: 5 6-OHDA:5
7*L*	(1) One-way ANOVA: *F*_(3, 14)_ = 7.106, *p* = 0.0039** (2) Two-way ANOVA: Time × Contra/Ipsi: *F*_(3, 14)_ = 1.674, *p* = 0.2179 Time: *F*_(3, 14)_ = 8.620, *p* = 0.0017** Contra/Ipsi: *F*_(1, 14)_ = 6.461, *p* = 0.0235*	Bonferroni's multiple-comparisons test 0 vs 1 week: *p* = 0.0064^++^ 0 vs 4 weeks: *p* > 0.9999 0 vs 8 weeks: *p* > 0.9999 Bonferroni's multiple-comparisons test 0, contra vs ipsi: *p* > 0.9999 1 week, contra vs ipsi: *p* = 0.0114* 4 weeks, contra vs ipsi: *p* = 0.6686 8 weeks, contra vs ipsi: *p* > 0.9999	Naïve: 3 1 week: 7 4 weeks: 4 8 weeks: 4
7*M*	(1) One-way ANOVA: *F*_(3, 13)_ = 9.003, *p* = 0.0017** (2) Two-way ANOVA: Time × Contra/Ipsi: *F*_(3, 13)_ = 4.561, *p* = 0.0215* Time: *F*_(3, 13)_ = 4.057, *p* = 0.0308* Contra/Ipsi: *F*_(1, 13)_ = 9.421, *p* = 0.0090**	Bonferroni's multiple-comparisons test 0 vs 1 week: *p* = 0.0135^+^ 0 vs 4 weeks: *p* = 0.0531 0 vs 8 weeks: *p* > 0.9999 Bonferroni's multiple-comparisons test 0, contra vs ipsi: *p* > 0.9999 1 week, contra vs ipsi: *p* = 0.0110* 4 weeks, contra vs ipsi: *p* = 0.0589 8 weeks, contra vs ipsi: *p* > 0.9999	Naïve: 4 1 week: 4 4 weeks: 4 8 weeks: 5
7*O*	Two-way ANOVA: Contra/Ipsi × Saline/6-OHDA: *F*_(1, 7)_ = 4.165, *p* = 0.0806 Contra/Ipsi: *F*_(1, 7)_ = 0.2406, *p* = 0.6387 Saline/6-OHDA: *F*_(1, 7)_ = 6.297, *p* = 0.0404*	Bonferroni's multiple-comparisons test Contra, Saline vs 6-OHDA: *p* > 0.9999 Ipsi, Saline vs 6-OHDA: *p* = 0.0123*	Saline: 5 6-OHDA:4
7*P*	Two-way ANOVA: Contra/Ipsi × Saline/6-OHDA: *F*_(1, 7)_ = 0.6895, *p* = 0.4337 Contra/Ipsi: *F*_(1, 7)_ = 6.132, *p* = 0.0424* Saline/6-OHDA: *F*_(1, 7)_ = 1.655, *p* = 0.2391		Saline: 5 6-OHDA:4
8*C*	Mann-Whitney Cre- vs Cre+, *p* = 0.0002***		Pirt Cre-ChR2 flox/+: 8 Pirt Cre + ChR2 flox/+: 7
8*D*	Two-way ANOVA: Time × Sham/SNI: *F*_(8, 144)_ = 32.47, *p* < 0.0001**** Time: *F*_(3.946, 71.03)_ = 32.47, *p* < 0.0001**** Sham/SNI: *F*_(1, 18)_ = 113.2, *p* < 0.0001****	Bonferroni's multiple-comparisons test 0, sham vs SNI: 3 d, sham vs SNI: *p* < 0.0001**** 7 d, sham vs SNI: *p* < 0.0001**** 10 d, sham vs SNI: *p* < 0.0001**** 14 d, sham vs SNI: *p* < 0.0001**** 17 d, sham vs SNI: *p* = 0.0005*** 21 d, sham vs SNI: *p* = 0.0193* 24 d, sham vs SNI: *p* = 0.0119* 28 d, sham vs SNI: *p* = 0.3825	Pirt Cre + ChR2 flox/+, Sham: 9 Pirt Cre + ChR2 flox/+, SNI: 11
8*E*	Two-way ANOVA: Time × Sham/SNI: *F*_(8, 144)_ = 22.34, *p* < 0.0001**** Time: *F*_(3.623, 65.22)_ = 22.82, *p* < 0.0001**** Sham/SNI: *F*_(1, 18)_ = 753.8, *p* < 0.0001****	Bonferroni's multiple-comparisons test 0, sham vs SNI: 3 d, sham vs SNI: *p* < 0.0001**** 7 d, sham vs SNI: *p* < 0.0001**** 10 d, sham vs SNI: *p* < 0.0001**** 14 d, sham vs SNI: *p* < 0.0001**** 17 d, sham vs SNI: *p* < 0.0001**** 21 d, sham vs SNI: *p* = 0.0003*** 24 d, sham vs SNI: *p* = 0.0015** 28 d, sham vs SNI: *p* = 0.0002***	Pirt Cre + ChR2 flox/+; Sham: 9 Pirt Cre + ChR2 flox/+; SNI: 11
8*F* (pinprick)	RM One-way ANOVA: *F*_(4.068, 36.61)_ = 33.55, *p* < 0.0001****	Bonferroni's multiple-comparisons test 0 vs 3 d: *p* < 0.0001**** 0 vs 6 d: *p* < 0.0001**** 0 vs 8 d: *p* < 0.0001**** 0 vs 10 d: *p* < 0.0001**** 0 vs 13 d: *p* = 0.0004*** 0 vs 15 d: *p* < 0.0045** 0 vs 17 d: *p* < 0.0076** 0 vs 20 d: *p* = 0.0086** 0 vs 22 d: *p* = 0.6513 0 vs 24 d: *p* = 0.1559 0 vs 27 d: *p* > 0.9999	C57Bl6: 10
8*F* (1.4 g)	RM One-way ANOVA: *F*_(3.317, 29.85)_ = 33.26, *p* < 0.0001****	Bonferroni's multiple-comparisons test 0 vs 3 d: *p* < 0.0001**** 0 vs 6 d: *p* < 0.0001**** 0 vs 8 d: *p* < 0.0001**** 0 vs 10 d: *p* < 0.0001**** 0 vs 13 d: *p* < 0.0001**** 0 vs 15 d: *p* < 0.0001**** 0 vs 17 d: *p* < 0.0001**** 0 vs 20 d: *p* = 0.0249* 0 vs 22 d: *p* = 0.3009 0 vs 24 d: *p* = 0.8112 0 vs 27 d: *p* > 0.9999 0 vs 29 d: *p* = 0.1795	C57Bl6: 10
8*G*	Two-way ANOVA: Force × Time: *F*_(21, 216)_ = 8.710, *p* < 0.0001**** Force: *F*_(7, 72)_ = 47.92, *p* < 0.0001**** Time: *F*_(2.454, 176.7)_ = 88.19, *p* < 0.0001****	Bonferroni's multiple-comparisons test Baseline vs SNI 1 week: *p* < 0.0001**** Baseline vs SNI 4 weeks: *p* = 0.7396 Baseline vs SNI 8 weeks: *p* > 0.9999	C57Bl6: 10
8*H*	Two-way ANOVA: Force × Time: *F*_(21, 216)_ = 3.028, *p* < 0.0001**** Force: *F*_(7, 72)_ = 18.49, *p* < 0.0001**** Time: *F*_(2.688, 193.5)_ = 67.76, *p* < 0.0001****	Bonferroni's multiple-comparisons test Baseline vs SNI 1 week: *p* < 0.0001**** Baseline vs SNI 4 weeks: *p* < 0.0001**** Baseline vs SNI 8 weeks: *p* < 0.0001****	C57Bl6: 10
8*I*	Two-way ANOVA: Time × Contra/Ipsi: *F*_(8, 144)_ = 5.811, *p* < 0.0001**** Time: *F*_(4.399, 79.19)_ = 2.187, *p* = 0.0719 Contra/Ipsi: *F*_(1, 18)_ = 13.56, *p* = 0.0017**	Bonferroni's multiple-comparisons test 0, Contra vs Ipsi: *p* > 0.9999 1 week, Contra vs Ipsi: *p* < 0.0001**** 2 weeks, Contra vs Ipsi: *p* = 0.0054** 3 weeks, Contra vs Ipsi: *p* = 0.0121* 4 weeks, Contra vs Ipsi: *p* > 0.9999 5 weeks, Contra vs Ipsi: *p* > 0.9999 6 weeks, Contra vs Ipsi: *p* > 0.9999 7 weeks, Contra vs Ipsi: *p* > 0.9999 8 weeks, Contra vs Ipsi: *p* > 0.9999	C57Bl6: 10

*^,^^+^*p* < 0.05; **^,^^++^*p* < 0.01; ***^,^^+++^*p* < 0.001; ****^,^^++++^*p* < 0.0001.

#### von Frey assay

Mice were placed under ventilated plexiglass boxes on a wire mesh platform and habituated for at least 2 h per day for at least 2 d prior to the experiment. On the test day, mice were habituated for at least 30 min before the assay. A series of von Frey filaments (North Coast Medical NC12775-02 to NC12775-09) were applied perpendicularly to the point of bending to either the hindpaw sural nerve-innervated plantar skin or the hindpaw tibial nerve-innervated plantar hairy skin located between the footpads. The nominal bending forces of the filaments, provided by the manufacturer, were 0.02, 0.04, 0.07, 0.16, 0.4, 0.6, 1, and 1.4 g. Paw withdrawal or flinching immediately upon filament application was defined as a positive response. For each given force, the filament was applied 5 times to the ipsilateral (i.e., on side of injury) hindpaw. In some cases, the filament was applied 5 times to the contralateral hindpaw, then applied 5 times to the ipsilateral hindpaw. Intervals between each application were at least a few seconds to avoid sensitization. The number of positive responses out of 5 total applications was used to calculate a given animal's response frequency.

#### Pinprick assay

This assay was generally performed at least 30 min after the von Frey assay was completed, on the same platform. An Austerlitz insect pin (000, Fine Scientific Tools) was applied to the hindpaw plantar hairy skin. Paw withdrawal or flinching immediately upon filament application was defined as a positive response. Only the ipsilateral hindpaw was tested. Intervals between each application were at least a few seconds to avoid sensitization. The number of positive responses out of 5 total applications was used to calculate a given animal's response frequency.

#### Optogenetics

Mice were placed under an inverted 500 ml beaker on a glass platform and habituated for at least 2 h per day for at least 2 d prior to the actual experiment. On the test day, mice were habituated for at least 30 min before the assay. A 473 nm Blue DPSS laser (Laserglow technologies, Canada) was used to optogenetically stimulate nerve fibers (Browne et al., 2017). 10–100 ms light pulses were applied precisely to the tibial area of the plantar surface of the hindpaw using an optic fiber cable (Ø400 μm core, M82L01, Thorlabs, Inc., USA). Intervals between each application were at least a few seconds to avoid sensitization. Cre-negative and Cre-positive sham mice were used as controls. The number of positive responses out of 5 total applications was used to calculate a given animal's response frequency.

### 6-Hydroxydopamine injection

For systemic injection, mice received intraperitoneal injections of 6-OHDA (100 mg/kg, Santa Cruz) in saline for 5 consecutive days either at 8 weeks after SNI, or without prior injury. Control mice received intraperitoneal injections of saline. For local injection, under deep isoflurane anesthesia, mice received an injection of 6-OHDA (2 μl, 100 μg/μl) or saline, delivered via a Hamilton syringe connected to a 30G needle, into the hindpaw plantar hairy skin for 5 d.

### Fast blue retrograde labeling

Mice were anesthetized using isoflurane and 1–2 μl of FB (1% in saline; Polysciences Inc.) was injected subcutaneously using a Hamilton syringe connected to a 30-gauge needle to label sensory afferents that innervate the injection sites. Retrogradely labeled sensory neurons in DRGs were examined 7 d after injection. To evaluate the subtypes of neurons exhibiting collateral sprouting, FB was injected at the time of SNI surgery or at 7 weeks postoperatively.

### 4-Hydroxytamoxifen injections

4-hydroxytamoxifen (4-HT, Sigma-Aldrich) was dissolved in 100% ethanol (10 mg/ml), mixed with wheat germ oil (Jedwards International Inc., USA), vortexed for 1 min and centrifuged under vacuum for 20–30 min to remove the ethanol. For TH^2ACreER^ animals, 100 μl (1 mg) of the 4-HT solution was delivered via oral gavage at P13, P14 and P15. For TrkC^CreER^ animals, 20 μl (0.1 mg) of the 4-HT solution was delivered via intraperitoneal injection at P5.

### Immunohistochemistry

Mice were transcardially perfused with phosphate-buffered saline (PBS) followed by 4% paraformaldehyde (PFA) in PBS and L3–L5 dorsal root ganglia (DRG) and hindpaw skin were harvested. For transverse tissue section preparation, tissues were postfixed in 4% PFA at 4°C overnight. Tissues were cryoprotected in 30% sucrose in phosphate buffer at 4°C overnight, embedded in optimal cutting temperature medium (OCT, Tissue-Tek) and stored at −80°C. Tissues were cryostat sectioned at 10 µm for DRG and 16 µm for hindpaw skin, respectively. DRG and skin sections were thaw-mounted onto glass slides, stored at −80°C, and incubated at 30–37°C for 20 min immediately prior to staining. Slides sections were washed with 0.1% Triton X-100 in PBS (PBST.1) 3x 10 min. Slides sections were then blocked with 0.3% Triton X-100 in PBS containing 10% normal donkey serum for 1 h at room temperature. Tissues were incubated overnight with primary antibodies against rat anti-K8 (Univ of Iowa/DSHB, 1:100, #Troma-1), rabbit anti-K17 (from Dr. Pierre Coulombe Univ of Michigan, 1:1000), Chicken anti-NFH (Aves Labs, 1:200, #NFH), goat anti-mCherry (Sicgen, 1:500, #AB0040-500), chicken anti-GFP (Aves Labs, 1:400, #GFP-1020), goat anti-GFP (Sicgen, 1:500, #AB0020), rabbit anti CGRP (ImmnuoStar, 1:1000, #24112), Goat anti-GFRα2 (R&D Systems, #AF429), goat anti CGRP (Abcam, 1:500, #AB36001), Biotin-IB4 (Sigma, 1:100, #L2140), sheep anti-TH (EMD Millipore, 1:500, #AB1542), rabbit anti-TH (EMD Millipore, 1:500, AB152), and chicken anti-NeuN (Aves Labs, 1:200, #NUN), at room temperature in a humidity chamber. The following day tissues were washed with PBST.1 3x 10 min and then incubated for 1–2 h at room temperature in a humidity chamber with secondary antibodies; donkey anti-goat Cy3 (Jackson ImmunoResearch, 1:500, #705-166-147), goat anti-chicken 546 (Thermo Fisher, #A11040), donkey anti-rabbit 647 (Jackson ImmunoResearch, #711-605-152), goat anti-rat 488 (Jackson ImmunoResearch, # 112-545-003), donkey anti-chicken 488 (Jackson ImmunoResearch, #703-545-155), donkey anti-rat 488 (Jackson ImmunoResearch, #712-545-153), goat anti-chicken 488 (Thermo Fisher, #A11039), goat anti-rat Cy3 (Jackson ImmunoResearch, #112-165-167), donkey anti-chicken Cy3 (Jackson ImmunoResearch, #703-165-155), donkey anti-goat 557 (R&D Systems, #NL001), and streptavidin-Dylight 405 (Thermo Fisher, #21831). Tissues were then washed with PBS 3x 10 min. Floating spinal cord sections were rinsed in water or 0.1 M phosphate buffer, mounted on slides, and allowed to air dry. Sections were coverslipped using Dako fluorescence mounting medium (Dako, #S3023).

For whole-mount hindpaw skin staining, fat and connective tissue were thoroughly removed to facilitate antibody penetration. Tissues were postfixed in 4% PFA at 4°C overnight and then briefly washed with PBS to remove excess PFA. Tissues were washed with 1% Triton X-100 in PBS (PBST.hi) 10 × 30 min for a total of 5 h. Tissues were then incubated with primary antibodies diluted in blocking solution (75% PBST.hi, 20% DMSO, 5% normal donkey/goat serum) for 3 d at room temperature. Tissues were washed with PBST.hi 10x 30 min and then incubated with secondary antibodies diluted in blocking solution for 2 d at room temperature. Tissues were then again washed with PBST.hi 10 × 30 min and dehydrated in serial dilutions of MeOH (50%, 80%, 100% MeOH for 5 min each, and an additional 100% MeOH for 20 min). Finally, tissues were cleared in BABB (1 volume Benzyl Alcohol to 2 parts Benzyl Benzoate) for 30 min and mounted onto slides with BABB. All incubations were done on a rotating/rocking platform.

### In vivo electrophysiology

In vivo DRG electrophysiology recording was performed as previously described (Qu and Caterina, 2016). Briefly, Split^Cre^-Rosa26^LSLtdTomato^ mice were deeply anesthetized using chloral hydrate (500 mg/kg). A lateral laminectomy was performed to expose the lumbar DRGs, and oxygenated artificial cerebrospinal fluid (ACSF) was applied to the DRGs in the pool formed by stitching the incised skin into a ring. ACSF contained 130 mM NaCl, 3.5 mM KCl, 24 mM NaHCO_3_, 1.25 mM NaH_2_PO_4_, 1.2 mM MgCl_2_, 1.2 mM CaCl_2_, and 10 mM dextrose, was bubbled with 95% O_2_ and 5% CO_2_ and had a pH of 7.4. Extracellular recordings were obtained from individual sensory neurons whose epineurium was removed and labeled with tdTomato, using a polished suction micropipette. Receptive fields (RFs) of neurons innervating the hindpaw of the mouse were identified by probing the skin with a blunt glass probe. Mechanical stimuli were delivered to RFs innervated by DRG neurons, with a 0.5 mm diameter tipped probe using force-controlled indenter (Model 300C-I, Aurora Scientific). Conduction velocity (CV) was obtained by electrically stimulating the skin within the RF using two wire electrodes.

### Image analysis

Images were acquired using a confocal microscope (Nikon A1) and analyzed blinded to surgical time point using NIS elements (Nikon). For hindpaw skin whole-mount staining, Z-stack images (approximately 150–200 μm in total depth) were acquired across the thickness of the skin tissue. The tibial area of the glabrous skin of the hindpaw was defined as 1,200 μm × 1,272 μm (width × length) starting from a point 500 μm away from the medial hairy-glabrous border. The sural area of the glabrous skin in the hindpaw was defined as 800 μm × 1,272 μm in width and length beginning from the boundary of the lateral hairy skin. Hairy plantar skin was defined as the plantar skin region containing hair follicles and bounded by paw pads. For calculation of nerve fiber density from CGRP, NF-H, and TH staining, individual optical slices were subjected to background subtraction using the rolling ball correction tool, at a setting of 7.46 microns, a value empirically determined to eliminate a significant fraction of nonnerve fiber signal, while largely preserving fiber morphology. The resulting optical sections were compiled into maximum projection Z-stacks. Images were thresholded using a single value applied across all images of a given marker and a binary mask was created to define the regions with signal above threshold. The nerve density fraction was then calculated for each fluorescent channel as (area with signal above threshold)/(total area analyzed). In glabrous skin, the entire area was analyzed. In plantar hairy skin, to avoid counting signal from hair shaft autofluorescence, a binary mask corresponding to the visible hair shafts was manually traced and excluded from both the numerator and denominator areas for nerve density calculations. For quantification of tdTomato signal in TrkC^tdTomato^ and Split^Cre^;Rosa26^LSLtdTomato^ lines, quantification of nerve fiber density was the same with two exceptions. First, due to variability in overall staining intensity between tissue batches, thresholds were set for individual contralateral hindpaw images, blinded to timepoint, and then applied to the corresponding ipsilateral images from the same mice. Second, for Split^Cre^;Rosa26^LSLtdTomato^ mice, areas of cross-reacting cells with clear non-neuronal morphology were excluded from both the numerator and denominator, as described above for hair shafts. Figures shown throughout the manuscript were subjected only to threshold adjustment, without rolling ball background correction. The number of lanceolate or circumferential endings formed by labeled fibers in Split^Cre^;Rosa26^LSL-tdTomato^ or TH^2ACreER^;Rosa26^LSL-EYFP^ mice was counted manually by scrolling through the Z stacks of hairy plantar skin. For three-dimensional reconstructions of CGRP and NF-H colocalization, as well as TrkC and NF-H colocalization in plantar hairy skin, Z-stacks of images were acquired in 0.95 μm sections across the thickness of the skin tissue using a 16x water immersion objective and converted to three-dimensional images with IMARIS 10.1 software (Bitplane). Volumes of co-localization of markers within the image stacks were defined and quantified as a percent of total volume of staining for a given marker. For hindpaw transverse section staining, CGRP and GFRα2 intraepithelial nerve fiber (IENF) density per epidermal length was counted in at least 5 sections. Only the number of fibers crossing the dermal-epidermal boundary were counted, not multiple branches of the same fiber. Values for all parameters were expressed either as fractions within a given skin territory, numbers per unit skin area, or numbers per unit skin length, with each symbol shown in figures derived from an individual mouse. For DRG staining, neuronal cell type specific markers were counted as a control for total number of neurons. Approximately 900 neurons per mouse, derived from multiple sections were counted for each data point. The number of FB positive or FB/markers double-positive cells was counted in a blinded manner.

### Experimental design and statistical analysis

Full statistical results, including numbers of animals used in each experiment, are included in Table 1. Anatomical studies on C57BL6 mice and behavioral studies were performed on male mice. Anatomical studies on genetically labeled mice were performed on mice of both sexes. For immunostaining, when comparing only two groups, two-tailed Student's *t* test was used for analysis. When comparing only the ipsilateral hindpaw across multiple time points, one-way ANOVA was used. When comparing ipsilateral versus contralateral hindpaw across multiple time points, two-way ANOVA was used. For von Frey and pinprick behavioral measurements, when comparing only the ipsilateral hindpaw across multiple time points, repeated measures one-way ANOVA was used. When comparing ipsilateral versus contralateral hindpaw across multiple time points, repeated measures two-way ANOVA was used. For optogenetic assays, when comparing genotypes, Mann-Whitney test was used for analysis. Repeated measures two-way ANOVA was used to analyze the effects of group and/or time. ANOVA tests were followed by post hoc Bonferroni’s multiple-comparisons correction for either multiple times or multiple forces, but not both, in a given comparison. In repeated measures ANOVAs, potential differences related to sphericity were corrected for using the Geisser and Greenhouse method. All data were presented as mean ± SEM and the criterion for statistical significance was *p*-value <0.05. The exact statistical test used for each experiment and its details can be found in the figure legends and in Table 1. In the case of ANOVA analyses, *p*-values for the overall comparisons between groups are listed on the graphs, and *p* values at time points derived from the Bonferroni corrections are indicated by asterisks, as defined in the figure legends. Correlation coefficients were calculated using the Pearson correlation method, with correlation coefficient (*r*) ranging from −1 to +1. All analyses were performed using GraphPad Prism 9.

## Results

### Peripheral nerve injury leads to collateral sprouting of intact sensory nerves

**Figure 1.**
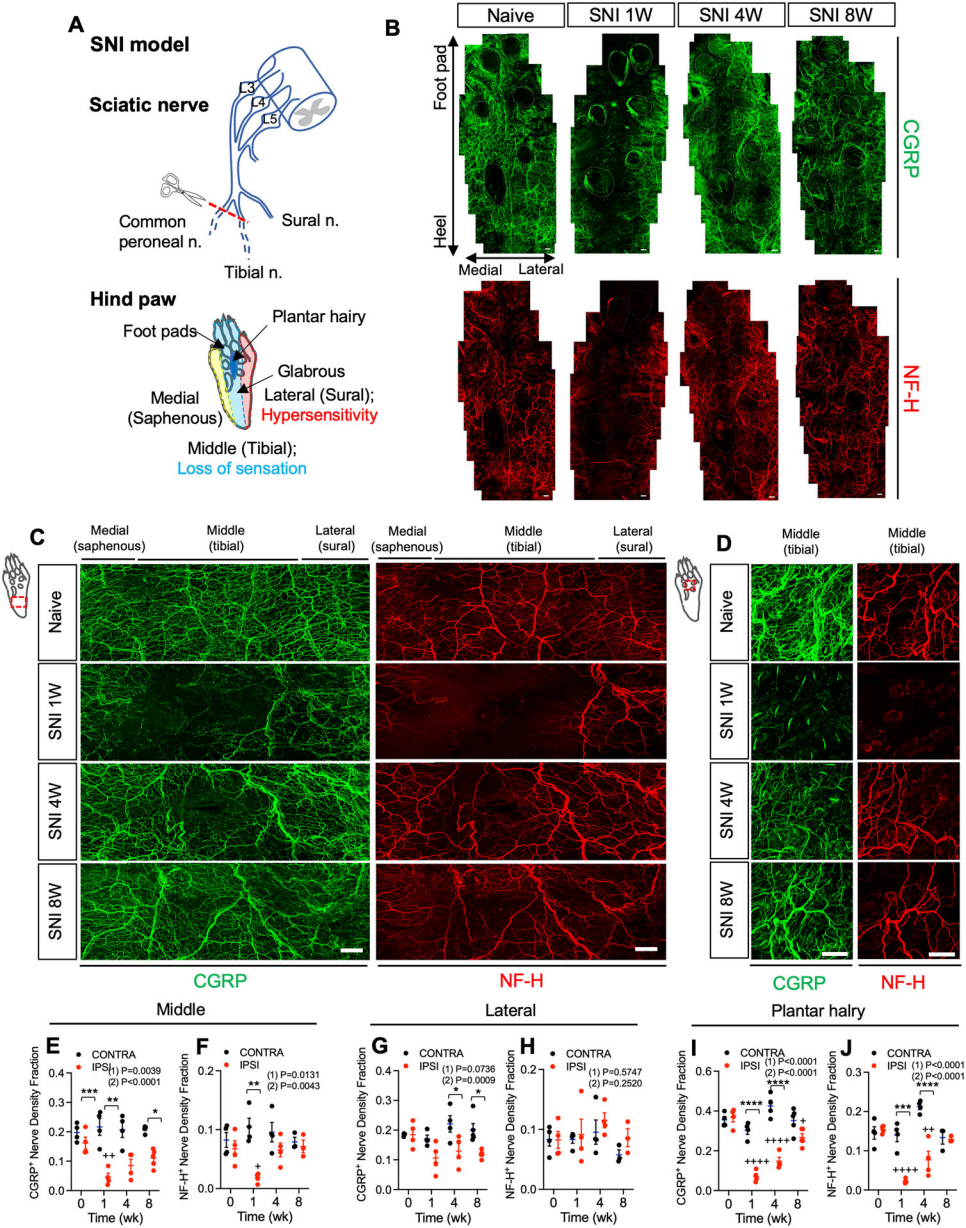
Collateral sprouting of spared nerve fibers after peripheral nerve injury. ***A***, Schematic of the SNI injury model, showing three branches of the sciatic nerve (common peroneal, tibial, and sural) and designating the lateral plantar region (red) of the hindpaw innervated by the sural nerve, the middle plantar region of the hindpaw innervated by the tibial nerve (blue), and the medial plantar region (yellow) of the hindpaw, which is innervated by the saphenous nerve. The dark blue region between the foot pads shows the plantar hairy skin area. ***B***, Tiled images of hindpaw plantar skin (digits omitted) following whole-mount immunostaining for CGRP (green) and NF-H (red) before and 1, 4, and 8 weeks after SNI. Footpad skin was not imaged. Scale bar, 100 μm. ***C***, Whole-mount immunostaining for CGRP (green) and NF-H (red) in the hindpaw glabrous skin of C57BL6 mice before and 1, 4, and 8 weeks after SNI surgery. Scale bar, 100 μm. ***D***, Whole-mount immunostaining for CGRP (green) and NF-H (red) in ipsilateral hindpaw plantar hairy skin before and 1, 4, and 8 weeks after SNI surgery. Scale bar, 100 μm. Residual green signal at 1 week is from hair shafts. ***E,F***, Quantification of CGRP (***E***) and NF-H (***F***) nerve density fraction in contralateral (black) and ipsilateral (red) middle area of hindpaw glabrous skin at the indicated times before and after SNI (*n* = 3–5). ***G,H***, Quantification of CGRP (***G***) and NF-H (***H***) nerve density fraction in contralateral (black) and ipsilateral (red) lateral area of hindpaw glabrous skin at the indicated times before and after SNI (*n* = 3–5). ***I,J***, Quantification of CGRP (***I***) and NF-H (***J***) nerve density fraction in contralateral (black) and ipsilateral (red) plantar hairy skin at the indicated times before and after SNI (*n* = 3–5). Data are presented as mean ± SEM. In panels ***E–H***, (1) indicates Overall *p*-value for difference between baseline and ipsilateral hindpaw s over time using one-way ANOVA. Results of Bonferroni post hoc correction shown as: ^+^*p* < 0.05; ^++^*p* < 0.01; ^+++^*p* < 0.001; ^++++^*p* < 0.0001. (2) Indicates overall *p*-value for difference between ipsilateral and contralateral paws over time using two-way ANOVA. Results of Bonferroni post hoc correction shown as: *p < 0.05; **p < 0.01; ***p < 0.001; ****p < 0.0001. Full statistical details can be found in Table 1.

To assay for collateral sprouting of intact nerves into denervated regions after nerve injury, we performed whole mount immunofluorescence (IF) staining of mouse hindpaw skin prior to and 1, 4, and 8 weeks following spared nerve injury (SNI) surgery (Fig. 1*A*). It has been shown in the rat SNI model that transected nerves do not regenerate past the lesion site (Xie et al., 2017). Thus, any reinnervation in this model is attributable to collateral sprouting. One week after SNI in the mouse, we observed a substantial loss of immuno-reactivity (IR) for peptidergic (CGRP^+^) and myelinated (NF-H^+^) nerve fibers in the middle of the ipsilateral hindpaw glabrous skin formerly supplied by the tibial nerve. However, at 4 and 8 weeks after injury, progressive reinnervation by CGRP^+^ and NF-H^+^ nerve fibers from medial (saphenous) and lateral (sural) territories into denervated glabrous skin was observed (Fig. 1*B,C*). These changes were quantified from 2D projections of confocal image stacks (Fig. 1*E,F*), and confirmed our prior qualitative impression of glabrous skin NF-H^+^ fiber loss and restoration after SNI surgery (Jeon et al., 2021).

**Figure 2.**
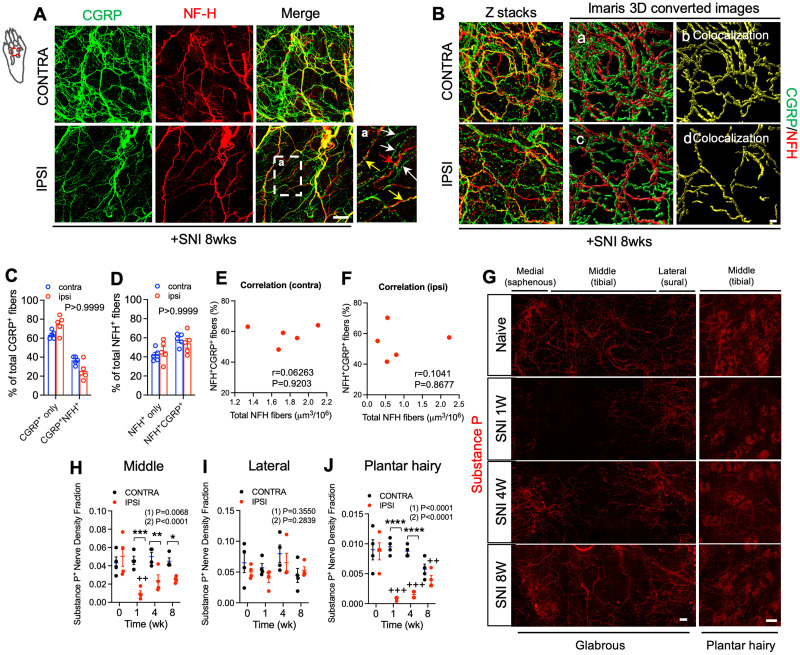
Correlation between CGRP and NF-H labeling in hindpaw skin nerve fibers after peripheral nerve injury and sprouting of Substance P labeled fibers. ***A***, Double immunostaining for CGRP (green) and NF-H (red) in the hindpaw 8 weeks after SNI. Inset (a) shows higher magnification view of the area indicated by dashed box. White arrows indicate CGRP^+^ nerve fibers, red arrows indicate NF-H^+^ nerve fibers, and yellow arrows indicate double-positive fibers, respectively. Scale bar, 100 μm. ***B***, Double immunostaining for CGRP^+^ (green) and NF-H^+^ (red) in hindpaw 8 weeks after SNI surgery. Subpanels a–d indicate IMARIS 3D rendered images. In subpanels b and d colocalization (yellow) of CGRP^+^ and NF-H^+^ is shown. Scale bar, 20 μm. ***C***, Quantification of the percentages of CGRP^+^ only and CGRP^+^NF-H^+^ fibers among total CGRP^+^ nerve fibers in contralateral and ipsilateral plantar hairy skin 8 weeks after SNI surgery (*n* = 5). ***D***, Quantification of the percentages of NFH^+^ only and NF-H^+^CGRP^+^ fibers among total NFH^+^ nerve fibers in contralateral and ipsilateral plantar hairy skin 8 weeks after SNI surgery (*n* = 5). ***E,F***, Correlation between percent of NF-H^+^CGRP^+^ fibers and total NFH^+^ nerve fiber density in contralateral (N) and ipsilateral (O) plantar hairy skin 8 weeks after SNI surgery (*n* = 5). ***G***, Whole-mount immunostaining for Substance P (red) in the hindpaw glabrous skin (left panel) and plantar hairy skin (right panel) of C57BL6 mice before and 1, 4, and 8 weeks after SNI surgery. Scale bar, 100 μm. ***H***, Quantification of Substance P nerve density fraction in contralateral (black) and ipsilateral (red) middle area of hindpaw glabrous skin at the indicated times before and after SNI (*n* = 4). ***I***, Quantification of Substance P nerve density fraction in contralateral (black) and ipsilateral (red) lateral area of hindpaw glabrous skin at the indicated times before and after SNI (*n* = 4). ***J***, Quantification of Substance P nerve density fraction in contralateral (black) and ipsilateral (red) plantar hairy skin at the indicated times before and after SNI (*n* = 4). Data are presented as mean ± SEM. In panels ***C*** and ***D***, overall *p*-value is from difference between ipsilateral and contralateral paws using two-way ANOVA. In panels ***E*** and ***F***, correlation coefficient (*r*) and *p*-value from Pearson correlation, NF-H^+^CGRP^+^ versus total NFH^+^. In panels ***H–J***, (1) indicates Overall *p*-value for difference between baseline and ipsilateral hindpaw s over time using one-way ANOVA. Results of Bonferroni post hoc correction shown as: ^+^*p* < 0.05; ^++^*p* < 0.01; ^+++^*p* < 0.001; ^++++^*p* < 0.0001. (2) Indicates overall *p*-value for difference between ipsilateral and contralateral paws over time using two-way ANOVA. Results of Bonferroni post hoc correction shown as: *p < 0.05; **p < 0.01; ***p < 0.001; ****p < 0.0001. Full statistical details can be found in Table 1.

**Figure 3.**
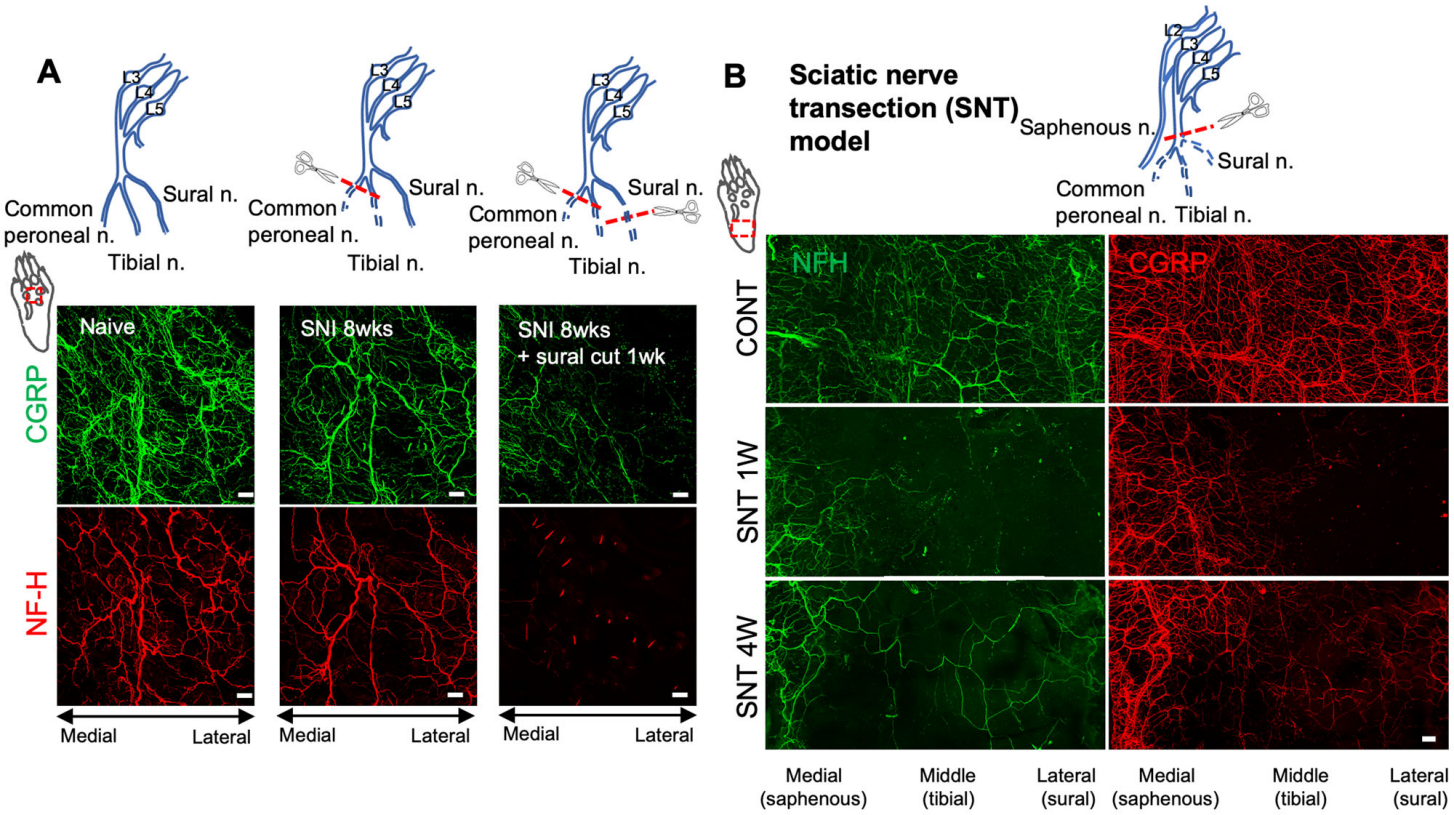
Collateral sprouting of intact saphenous nerve fibers after peripheral nerve injury. ***A***, Schematic showing the site of nerve damage (top panels) either following sural-sparing SNI surgery (middle diagram) or sural sparing SNI surgery, followed at 8 weeks by sural nerve transection (right diagram). Bottom panels show CGRP (green) and NF-H (red) immunostaining in plantar hairy hindpaw at indicated time points. Scale bar, 100 μm. ***B***, At top is schematic of the SNT injury model, showing saphenous nerve and three branches of the sciatic nerve. At bottom is whole-mount immunostaining for NF-H (green) and CGRP (red) in the hindpaw glabrous skin of C57BL6 mice 1 and 4 weeks after SNT surgery. Scale bar, 100 μm.

**Figure 4.**
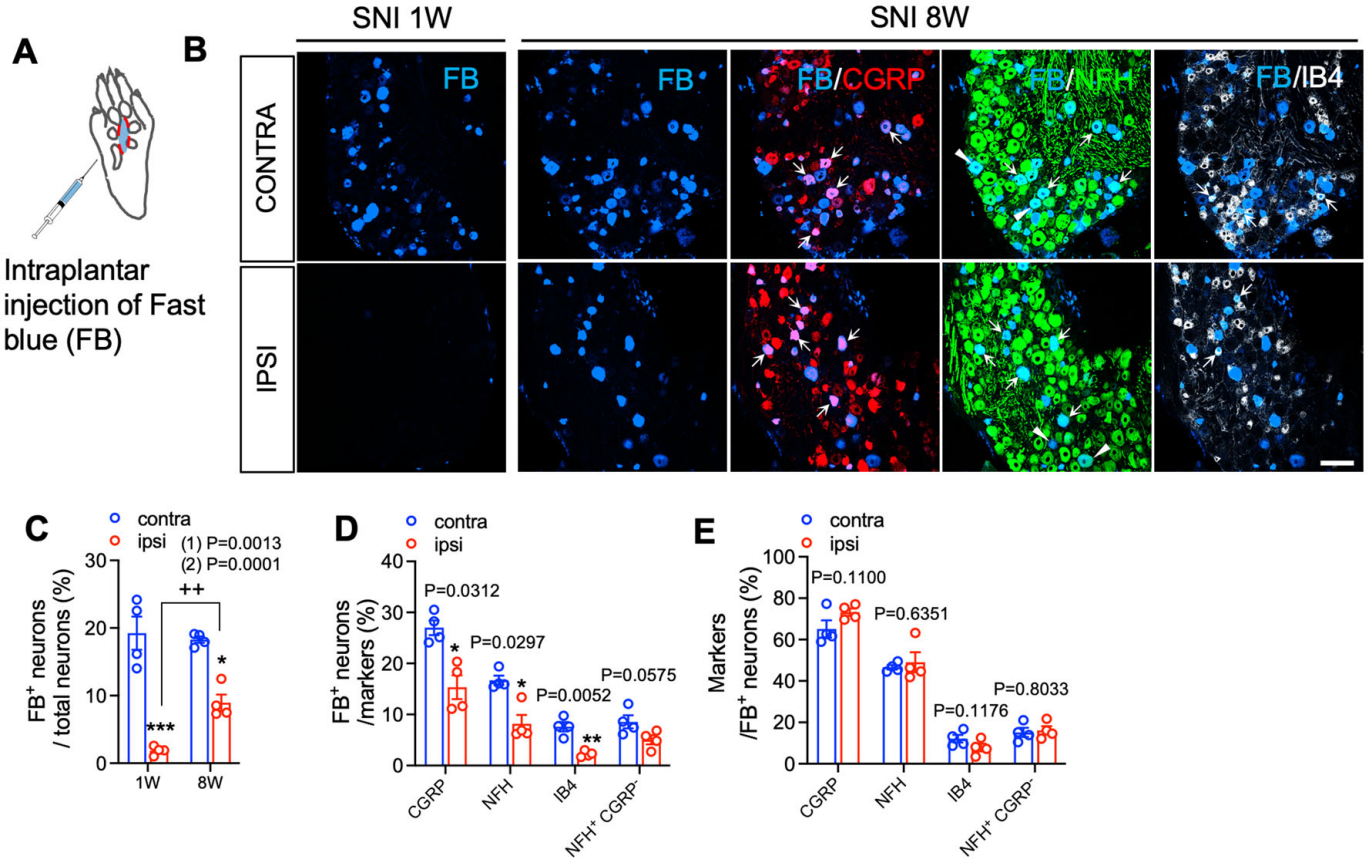
Subtypes of neurons exhibiting collateral sprouting after peripheral nerve injury. ***A***, Schematic of intraplantar injection of FB into the hindpaw plantar hairy skin. ***B***, FB retrogradely labeled plantar hairy skin-innervating neurons (blue) in the contralateral and ipsilateral lumbar DRGs of C57BL6 mice 1 (left panel) and 8 (right panel) weeks after SNI surgery. Immunostaining for CGRP (red), NF-H (green), and IB4 (white) with FB retrogradely labeled plantar hairy skin-innervating neurons (blue) in lumbar DRGs from C57BL6 mice 8 weeks after SNI surgery. Arrows indicate retrogradely labeled FB/markers doubly positive neurons. Pointed triangles indicate retrogradely labeled FB/NF-H^+^/CGRP^−^ neurons. Scale bar, 100 μm. ***C***, Quantification of the percentages of retrogradely labeled FB positive neurons in the contralateral (blue) and ipsilateral (red) lumbar DRGs of C57BL6 mice 1 and 8 weeks after SNI surgery (*n* = 4). ***D***, Quantification of the percentages of FB labeled neurons in CGRP^+^, NF-H^+^, IB4^+^, NF-H^+^ CGRP^−^ neurons in lumbar DRGs from C57BL6 mice 8 weeks after SNI surgery (*n* = 4). ***E***, Quantification of the percentages of CGRP^+^, NF-H^+^, IB4^+^, NF-H^+^ CGRP^−^ neurons in FB labeled neurons in lumbar DRGs from C57BL6 mice 8 weeks after SNI surgery (*n* = 4). Data are presented as mean ± SEM. In panel ***C***, (1) indicates unpaired two-tailed Student's *t* test. ^++^*p* < 0.01, 1 week versus 8 weeks. (2) Indicates overall *p*-value for difference between ipsilateral and contralateral paws over time using two-way ANOVA. Results of Bonferroni post hoc correction shown as: *p < 0.05; ***p < 0.001. In panels ***D*** and ***E***, *p*-values from paired two-tailed Student's *t* test are shown at top as: *p < 0.05, **p < 0.01, contra versus ipsi. Full statistical details can be found in Table 1.

Next, we quantified CGRP^+^ and NF-H^+^ nerve fibers within a region of the lateral hindpaw glabrous skin. This territory is supplied predominantly by the intact sural nerve, but could also have overlapping contributions from the tibial nerve. Indeed, a slight decrease in CGRP^+^ nerve fibers was observed in this lateral hindpaw skin territory that persisted until 8 weeks after SNI (Fig. 1*B,C,G*). However, no change in sural territory NF-H^+^ nerve fibers was observed at any time point (Fig. 1*B,C,H*). A third skin territory analyzed was that in the vicinity of a group of recently described small hairs between foot pads in the plantar skin of C57BL6 mice (Walcher et al., 2018; Jeon et al., 2021). In this plantar hairy skin, we again saw a significant reduction of CGRP^+^ and NF-H^+^ nerve fibers 1 week after injury. As in glabrous skin, however, innervation began to return by 4 weeks and had nearly fully recovered by 8 weeks (Fig. 1*B,D,I,J*). Because a subset of CGRP expressing sensory neurons are known to be neurofilament heavy chain-positive, and vice versa, we also examined the overlap between these two markers among reinnervating fibers in glabrous skin. Whereas a subset of fibers were positive for both markers, and are likely to represent myelinated peptidergic nociceptors (Usoskin et al., 2015; Ghitani et al., 2017), CGRP-only and NF-H-only fibers were also observed (Fig. 2*A*). We therefore measured the co-localization of CGRP and NF-H fibers after nerve injury from 3D image stacks using IMARIS software. The 3D rendered images showed that the relative abundance of CGRP-only and NF-H-only fibers as well as CGRP^+^NF-H^+^ fibers were no different from the contralateral paw skin (Fig. 2*B–D*). There was also no correlation between the percent of CGRP^+^NF-H^+^ double labeled fibers and the total density of NFH^+^ fibers in either ipsilateral or contralateral hindpaw after nerve injury (Fig. 2*E,F*). Some peptidergic nociceptors express a different neuropeptide, Substance P (SP) (Woolf and Ma, 2007; Basbaum et al., 2009). Immunostaining of glabrous and plantar hairy paw skin before and after nerve injury revealed that tibial- (but not sural-) territory SP^+^ fibers are also lost following SNI surgery, but that, like CGRP^+^ fibers, they partially recover by 8 weeks (Fig. 2*G–J*). To confirm whether reinnervation of tibial territory skin had arisen by collateral sprouting, rather than regeneration, in a subset of mice, we axotomized the sural nerve 8 weeks after sural-sparing SNI surgery. While sprouted fibers presumably originating from the more medial saphenous nerve were preserved, there was a loss of fibers on the lateral side of the plantar hairy skin, consistent with a sural nerve origin (Fig. 3*A*). As an additional test of this concept, we performed an alternative nerve injury surgery in mice, sciatic nerve transection (SNT), in which the sciatic nerve was transected and ligated proximal to its trifurcation, leaving only saphenous nerve innervation to the hindpaw skin. In this model, substantial loss of CGRP^+^ and NF-H^+^ nerve fibers was observed in both the tibial- and sural- territory glabrous skin at 1 week after nerve injury. However, reinnervation by both CGRP^+^ and NF-H^+^ nerves was observed 4 weeks after SNT, apparently indicative of sprouting from the intact saphenous nerve (Fig. 3*B*). As an independent means of evaluating the subtypes of neurons exhibiting collateral sprouting, we injected a small volume of the retrograde tracer dye, fast blue (FB) into the tibial territory hairy plantar skin of mice at the time of SNI surgery (Fig. 4*A*). Predictably, compared to the contralateral uninjured side, we observed an ∼4-fold reduction 1 week after surgery in the proportion of FB labeled cell bodies of lumbar dorsal root ganglion (DRG) neurons. By 8 weeks after denervation, however, the proportion of retrogradely labeled neurons on the injured side had recovered to approximately 50% of the contralateral control, consistent with these neurons projecting to the previously denervated skin (Fig. 4*B,C*). Examination of the subtypes of neurons exhibiting retrograde labeling 8 weeks after injury revealed that recovery was incomplete among CGRP^+^, NF-H^+^, and IB4^+^ (i.e., nonpeptidergic nociceptor/pruriceptor) populations (Fig. 4*B,D*). There was also a strong trend towards reduction of NFH^+^ but CGRP negative FB^+^ neurons that was not quite statistically significant (Fig. 4*D*). However, the relative abundance of each marker among those neurons exhibiting apparent sprouting was unchanged from control (Fig. 4*E*). Of note, these findings might be influenced by differential stoichiometries of cutaneous neuronal branch density per neuronal cell body among different subtypes and at different times during collateral sprouting.

**Figure 5.**
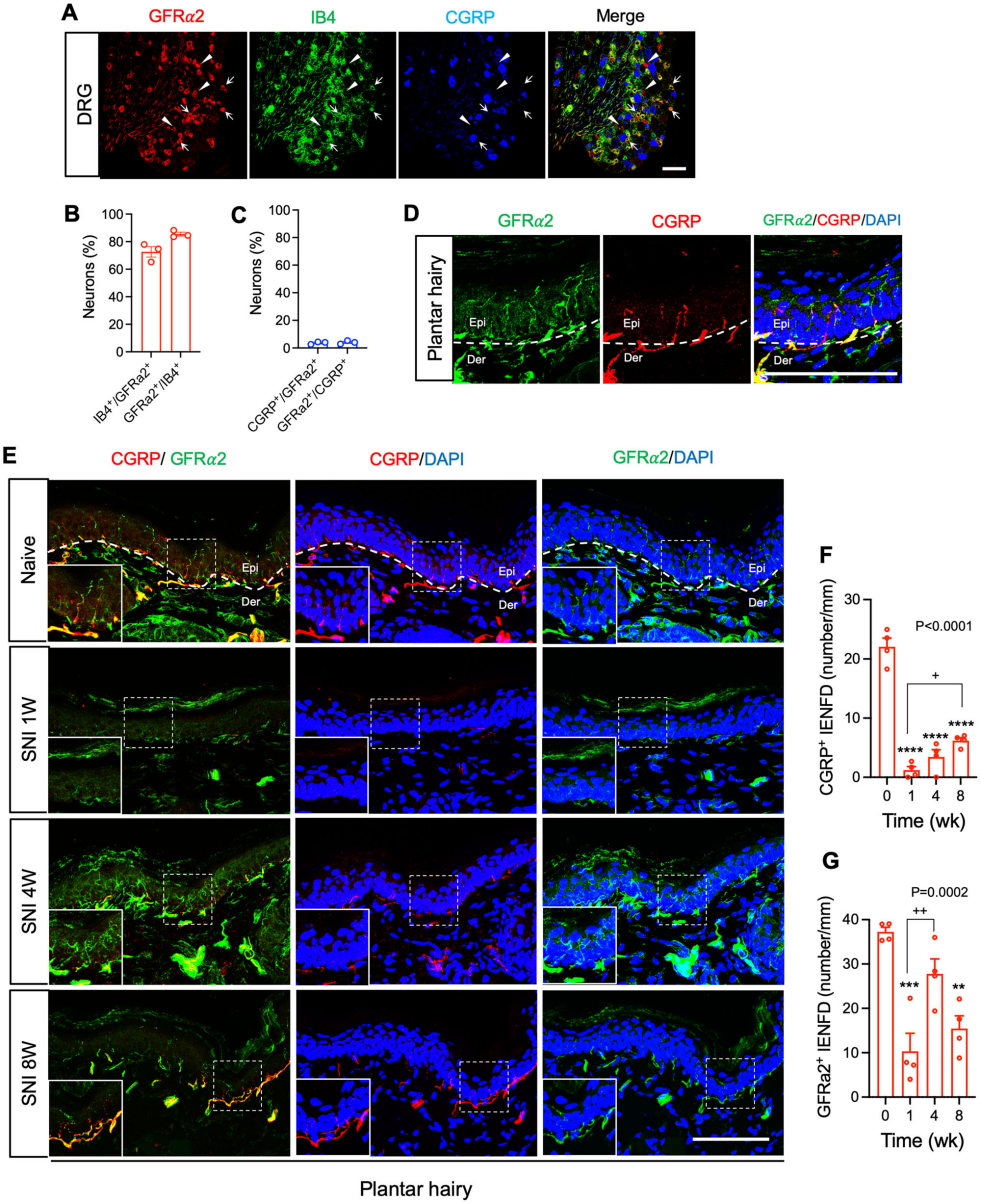
Epidermal reinnervation of peptidergic and nonpeptidergic spared nerve fibers after peripheral nerve injury. ***A***, Double immunostaining for GFRα2 (red), IB4 (green), and CGRP (blue) in lumbar DRGs from naïve C57BL6 mice. Arrows indicate neurons showing colocalization of GFRα2 and IB4. Pointed triangles indicate GFRα2 neurons not colocalizing with IB4. Scale bar, 100 μm. ***B***, Quantification of percentage of IB4^+^ neurons among total GFRα2^+^ neurons and of GFRα2^+^ neurons among total IB4^+^ neurons in lumbar DRG of C57BL6 mice (*n* = 3). ***C***, Quantification of percentage of strongly CGRP immunoreactive neurons among total GFRα2^+^ neurons and of GFRα2^+^ among strongly CGRP immunoreactive neurons in lumbar DRG of C57BL6 mice (*n* = 3). ***D***, Transverse section double immunostaining for GFRα2 (green) and CGRP (red) in the hindpaw hairy plantar skin from naïve C57BL6 mice. White dash is dermal-epidermal boundary. Scale bar, 100 μm. ***E***, Transverse section double immunostaining for CGRP (red) and GFRα2 (green) in the ipsilateral hindpaw hairy plantar skin before and 1, 4, and 8 weeks after SNI surgery. Curved white dash is dermal-epidermal boundary. Square insets show magnified view of CGRP-IENF and GFRα2-IENF. Scale bar, 100 μm. ***F,G***, Quantification of CGRP (***F***) and GFRα2 (***G***)-IENF density in the ipsilateral plantar hairy skin at the indicated times before and after SNI (*n* = 4). Data are presented as mean ± SEM. In panels ***F*** and ***G***, overall *p*-value is from one-way ANOVA of ipsilateral paw data over time. Results of Bonferroni post hoc correction shown as: **p < 0.01, ***p < 0.001, ****p < 0.0001, baseline versus ipsi; ^+^*p* < 0.05, ^++^*p* < 0.01, 1 week versus 4 weeks or 8 weeks. Full statistical details can be found in Table 1.

The analyses described above were based upon overall quantification of collateral sprouting into tibial territory skin, without consideration of the skin layers involved. We therefore next assessed intraepithelial nerve fiber loss and reinnervation by immunostaining transverse cryostat sections from hairy plantar skin for either the nonpeptidergic or peptidergic nociceptor/pruriceptor markers. Because IB4 binding exhibited a nonspecific pattern in skin, we instead used GFRα2 as a marker of nonpeptidergic nociceptor/pruriceptor terminals (Lindfors et al., 2006; Kupari and Airaksinen, 2014). We first confirmed that there was a substantial, but not exclusive, overlap between IB4 staining and GFRα2 staining among neuronal cell bodies in lumbar DRG (Fig. 5*A,B*). On the other hand, while GFRα2 was occasionally observed in neurons staining weakly for CGRP, it was rarely found in somata that stained strongly for CGRP (Fig. 5*A,C*). We further confirmed that epidermal terminal staining for CGRP and GFRα2 in plantar hairy skin of naïve mice was mostly, but not exclusively, nonoverlapping (Fig. 5*D*). As expected, 1 week following SNI surgery, we observed a dramatic decrease in both dermal and epidermal innervation by both CGRP^+^ and GFRα2^+^ fibers. Interestingly, at this time point, the disappearance of GFRα2 epidermal staining was not complete. Whether this represents incomplete/delayed degeneration of the transected nonpeptidergic fibers or a minor contribution of innervation by nonpeptidergic fibers of nontibial (e.g., sural or saphenous) origin is unclear. Nevertheless, we observed evidence of reinnervation by both populations, albeit with distinct kinetics. Reinnervation of the epidermis by CGRP^+^ fibers proceeded gradually and steadily from 1 to 8 weeks postinjury (Fig. 5*E,F*), but did not reach baseline levels in that timeframe. By contrast, reinnervation of epidermis by GFRα2^+^ fibers was profound at 4 weeks postinjury, achieving levels at or near baseline, but exhibited a trend towards regression between 4 and 8 weeks (Fig. 5*E,G*). These findings suggest that the forces driving and maintaining sprouting and epidermal penetration by peptidergic versus nonpeptidergic nociceptive/pruriceptive fibers following nerve injury are distinct.

### Differential collateral sprouting by Aβ rapidly adapting LTMRs versus TrkC lineage neurons

**Figure 6.**
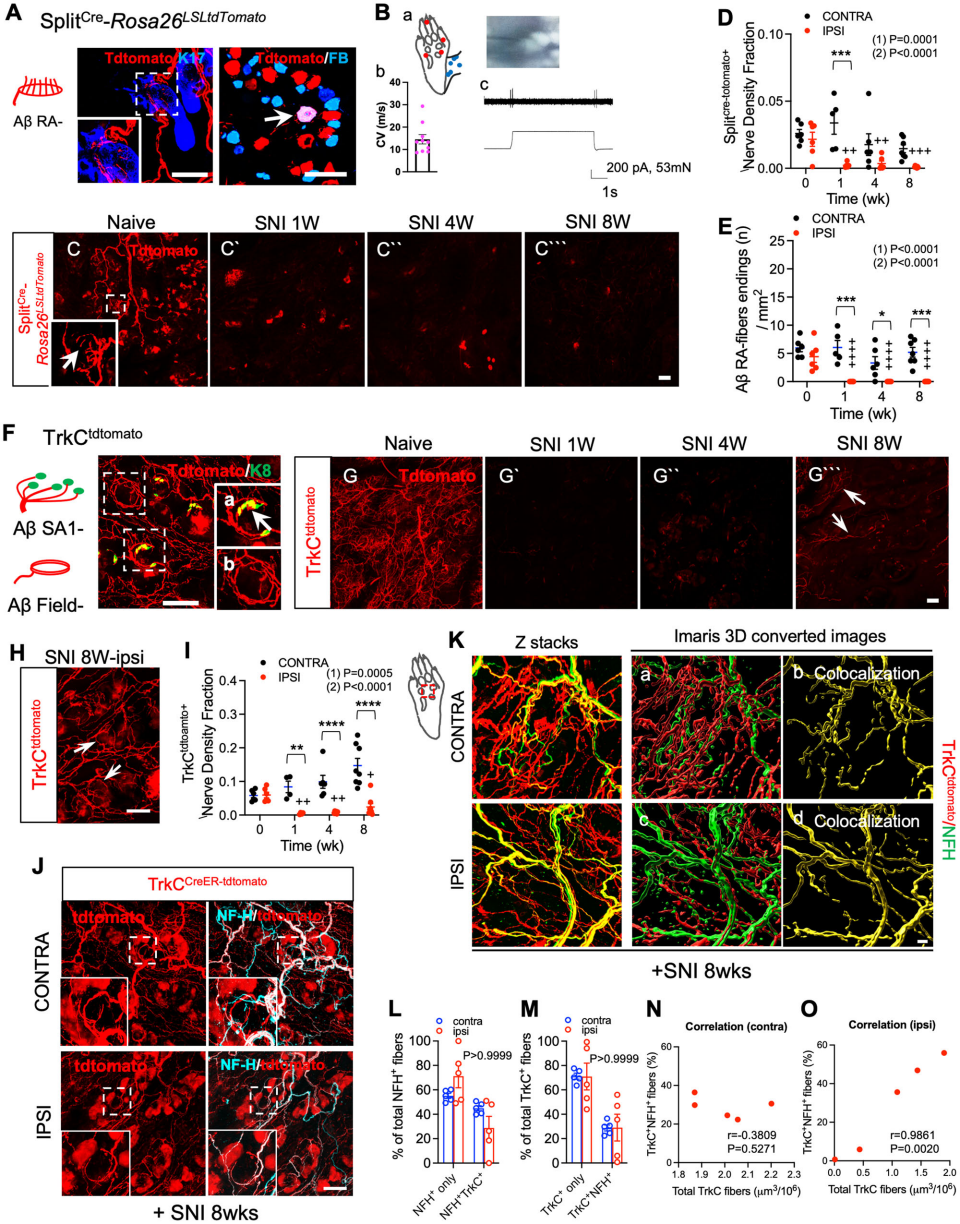
Aβ-LTMRs exhibit absent or limited sprouting into denervated skin after peripheral nerve injury. ***A***, Schematic of characteristic lanceolate endings of Aβ RA-LTMR labeled in Split^Cre^-Rosa26^LSLtdTomato^ mice (left). Modified from Abraira and Ginty (2013). Whole-mount immunostaining of plantar hairy skin from Split^Cre^-Rosa26^LSLtdTomato^ mice for tdTomato (red) and K17 (blue, hair follicle marker). Arrow in inset indicates lanceolate nerve ending (middle). Immunostaining for tdTomato (red) in FB retrogradely labeled plantar hairy skin-innervating neurons (blue) in lumbar DRGs from Split^Cre^-Rosa26^LSLtdTomato^. Arrow indicates retrogradely labeled FB/tdTomato doubly positive neurons (right). Scale bar, 100 μm. ***B***, In vivo electrophysiological recordings in the hindpaw from Split^Cre^-Rosa26^LSLtdTomato^ mice (*n* = 4). (a) Schematic showing receptive fields (RF) for all 10 individual neurons used for recording. Red dots represent neurons for which both mechanically stimulation response and conduction velocity (CV) were measured, and blue dots represent neurons for which only CV was measured. (b) A quantitative graph of CV of tdtomato^+^ neurons. (c) Representative in vivo electrophysiological recordings from an Aβ RA-LTMR in response to 80 mN mechanical stimulation with a 0.5 mm diameter tipped probe. ***C–C```***, In Split^Cre^-Rosa26^LSLtdTomato^ mice, whole mount immunostaining for tdTomato (red) in the ipsilateral plantar hairy skin of the hindpaw before (**C**) and 1(***C`***), 4(***C``***), and 8(***C```***) weeks after SNI. Arrow in inset indicates lanceolate nerve ending. Scale bar, 100 μm. ***D***, Quantification of Split^Cre^ Rosa26^LSL-tdTomato^ (*n* = 5–7) nerve density fraction in the contralateral (black) and ipsilateral (red) hindpaw plantar hairy skin at the indicated times after SNI. ***E***, Quantification of Split^Cre^-Rosa26^LSLtdTomato^ (Aβ RA-fiber, *n* = 5–7) tdTomato^+^ nerve fiber ending density in the contralateral (black) and ipsilateral (red) plantar hairy skin of hindpaw at the indicated times after SNI. ***F***, Whole-mount immunostaining of plantar hairy skin from TrkC^tdTomato^ mice for tdTomato (red) and K8 (green, Merkel cell). Insets show higher magnification views of the areas indicated by dashed boxes. Arrow in inset (a) indicates K8^+^ Merkel cells cluster and associated Aβ SA1 LTMR ending in plantar hairy skin. Inset (b) shows TrkC^tdTomato^ circumferential nerve ending, presumably a field LTMR. Scale bar, 100 μm. Images of tissues used in Figure 4*F* have been presented previously (Jeon et al., 2021). ***G–G```,H***, In TrkC^tdTomato^ mice, whole mount immunostaining for tdTomato (red) in the ipsilateral plantar hairy skin of the hindpaw before (***G***) and 1(***G`***), 4(***G``***), and 8(***G```***) weeks after SNI. Arrows indicate TrkC^tdTomato+^ fibers. In the ipsilateral hindpaw plantar hairy skin, arrows indicate TrkC^tdTomato^ circumferential nerve ending (***H***). Scale bar, 100 μm. ***I***, Quantification of TrkC^tdTomato^ (*n* = 5–8) nerve density fraction in the contralateral (black) and ipsilateral (red) hindpaw plantar hairy skin at the indicated times after SNI. ***J***, In TrkC^CreER^-Rosa26^LSL-EYFP^ mice with treated with 0.1 mg 4-HT at P5, whole mount immunostaining for tdTomato (red) in the ipsilateral plantar hairy skin of the hindpaw at 8 weeks after SNI. Insets indicate TrkC^tdTomato+^ fibers. Scale bar, 100 μm. ***K***, Double immunostaining for TrkC;tdTomato^+^ (red) and NF-H^+^ (green) in hindpaw 8 weeks after SNI. Subpanels a–d indicate IMARIS 3D rendered images. Subpanels b and d show colocalization (yellow) of TrkC;tdTomato^+^ and NF-H^+^ staining. Scale bar, 20 μm. ***L***, Quantification of the percentages of NF-H^+^ only and NF-H^+^ TrkC^+^ among total NF-H^+^ nerve fibers in contralateral and ipsilateral plantar hairy skin 8 weeks after SNI surgery (*n* = 5). ***M***, Quantification of the percentages of TrkC^+^ only and TrkC^+^ NF-H^+^ among total TrkC^+^ nerve fibers in contralateral and ipsilateral plantar hairy skin 8 weeks after SNI surgery (*n* = 5). ***N,O***, Correlation between percent TrkC^+^ NF-H^+^ double labeling and density of all TrkC^+^ nerve fibers in contralateral (***N***) and ipsilateral (***O***) plantar hairy skin 8 weeks after SNI surgery (*n* = 5). Data are presented as mean ± SEM. In panels ***D,E,I***, (1) indicates overall *p*-value for difference between baseline and ipsilateral hindpaw s over time using one-way ANOVA. Results of Bonferroni post hoc correction shown as: ^+^*p* < 0.05; ^++^*p* < 0.01; ^+++^*p* < 0.001; ^++++^*p* < 0.0001. (2) Indicates overall *p*-value for difference between ipsilateral and contralateral paws over time using two-way ANOVA. Results of Bonferroni post hoc correction shown as: *p < 0.05; **p < 0.01; ***p < 0.001; ****p < 0.0001. In panels ***K*** and ***L***, overall *p*-value is from difference between ipsilateral and contralateral paws using two-way ANOVA. In panels ***N*** and ***O***, correlation coefficient (*r*) and *p*-value from Pearson correlation; **p < 0.01, TrkC^+^ NF-H^+^ versus density of all TrkC^+^. Full statistical details can be found in Table 1.

We next turned to genetically labeled mouse lines to assess the potential of myelinated low-threshold mechanoreceptors (LTMRs), which also express neurofilament heavy chain, to exhibit collateral sprouting. The initial report of hair follicles in mouse hairy plantar skin described their innervation by Aδ-LTMRs and some circumferential fibers (Walcher et al., 2018). To more completely define the baseline LTMR innervation pattern of these hair follicles, we examined them in transgenic mice with selective labeling of additional LTMR subtypes (Fig. 6). We first examined Split^Cre^ Rosa26^LSL-tdTomato^ mice, in which Aβ RA-LTMRs are reportedly labeled (Rutlin et al., 2014). In naïve mice of this genotype, we observed tdTomato^+^ fibers and lanceolate endings surrounding hair follicles in plantar hairy skin (Fig. 6*A,C*). We also observed retrograde labeling of tdTomato^+^ cell bodies by FB injection into the plantar paw skin (Fig. 6*A*). Because limited physiological validation of the specificity of the Split^Cre^ Rosa26^LSL-tdTomato^ line has been reported (Rutlin et al., 2014), we conducted in vivo electrophysiological recording from the cell bodies of neurons labeled in these mice during controlled mechanical stimulation of the paw. We recorded from 10 tdTomato^+^ neurons in three mice. All 10 exhibited a conduction velocity in the Aβ range. Moreover, in the 4 neurons with mechanical receptive fields in the plantar paw skin, we observed a rapidly adapting response phenotype, including on- and off-responses, in all 4, consistent with their identity as Aβ-RA-LTMRs (Fig. 6*B*). We next examined TrkC^tdTomato^ mice, in which Aβ SA1-LTMRs and Aβ Field-LTMRs are labeled, in addition to other, less-well described populations (Li et al., 2011; Rutlin et al., 2014; Bai et al., 2015). As we previously reported (Jeon et al., 2021) we observed extensive innervation of hairy plantar skin with TrkC-lineage neurons that include Aβ SA1-LTMRs (Fig. 6*F*, arrow in inset a), defined by their association with Keratin 8 (K8)^+^ Merkel cells, and Aβ Field-LTMRs (Fig. 6*F*, inset b), defined by their circumferential endings. Thus, multiple Aβ- LTMR subtypes innervate plantar hairy skin under basal conditions. We also examined TrkB^CreER^-Rosa26^LSLtdTomato^ mice which, when treated postnatally with 4-hydroxytamoxifen, harbor labeled Aδ-LTMRs (Rutlin et al., 2014). However, while we observed cutaneous afferents in the hairy plantar hindpaw skin of these mice, extensive non-neuronal skin cell labeling (data not shown) precluded their effective use for subsequent experiments.

We then asked whether Aβ LTMR subtypes participate in collateral sprouting after nerve injury, by performing SNI surgery on labeled LTMR mouse lines. One week after injury, we observed an obvious reduction in nerve fibers in the plantar hairy skin of Split^Cre^ Rosa26^LSL-tdTomato^ (Fig. 6*C`,D*) and TrkC^tdtomato^ (Fig. 6*G`,I*) lines, compared to the contralateral paw (CONT). We also observed a reduction in lanceolate ending structures of Aβ RA-LTMR fibers in Split^Cre^ Rosa26^LSL-tdTomato^ mice (Fig. 6*C`,E*). Endings of Aβ SAI-LTMRs and Aβ-Field LTMRs, labeled in the TrkC^tdtomato^ mice, were not quantified but also appeared to be eliminated (Fig. 6*G`*). In contrast to our findings with nociceptive neurons, however, little or no convincing collateral sprouting of Aβ RA-LTMR fibers into the denervated region or formation of mature endings of these fibers was evident, even 8 weeks after SNI (Fig. 6*C```,D,E*). There was some residual tdTomato fluorescence 8 weeks after injury, but that signal did not resemble nerve fibers. These results suggest that unlike spared nociceptive neurons, spared Aβ RA-LTMRs fail to sprout into denervated skin after peripheral nerve injury, although we cannot definitively rule out trace amounts of sprouting. In TrkC^tdTomato^ mice, by comparison, we observed heterogeneous results at 8 weeks. In most mice, we observed a limited amount of tdTomato^+^ innervation. While such staining was consistently less than that seen in the contralateral paw (Fig. 6*G```*), some tdTomato^+^ fibers were evident in most mice. In one mouse (Fig. 6*H*), sprouting of TrkC positive fibers was robust, and included formation of circumferential endings as well as apparent innervation of sweat glands, although whether the latter was neuronal staining versus background was less clear. The existence of circumferential endings in the one animal suggests that they include Aβ-Field LTMRs. As a further confirmation of the sprouting of these fibers, we examined TrkC^CreER^ Rosa26^LSL-tdTomato^ mice which were injected postnatally with 4OHT. Prior studies have shown that postnatal activation of Cre in these mice results in labeling of fibers that include Aβ-Field LTMRs (Bai et al., 2015). At 8 weeks post SNI, tdTomato^+^ fibers were observed in plantar hairy skin of both contralateral and ipsilateral paws, including apparent circumferential endings that co-labeled with NF-H (Fig. 6*J*). We further analyzed the co-localization of the TrkC lineage marker and NF-H in hindpaw cutaneous fibers after nerve injury using IMARIS software. Analysis of 3D renderings of image stacks revealed that the relative abundance of TrkC-only, NF-H-only and TrkC^+^NF-H^+^ doubly labeled fibers were all indistinguishable between ipsilateral and contralateral plantar hairy skin (Fig. 6*K–M*). After nerve injury, there was also no correlation between the percent of TrkC^+^NF-H^+^ fibers and the total number of TrkC fibers in the contralateral hindpaw. However, after injury, there was a great deal of variability in the percentage of doubly positive fibers in the ipsilateral paw. Correspondingly, in the ipsilateral paw, we observed a strong positive correlation between these parameters, such that double labeling of the two markers was most prominent in mice exhibiting a greater extent of sprouting by TrkC lineage fibers (Fig. 6*N,O*). Together, these data suggest that in contrast to Aβ RA-LTMRs, which fail to sprout efficiently, some myelinated TrkC lineage fibers with endings morphologically resembling Aβ-Field LTMRs exhibit collateral sprouting capacity, albeit inconsistently. We cannot exclude, however, the possibility that these sprouted TrkC lineage fibers also include Aβ SAI-LTMRs, TrkC fibers formerly described to form free endings (Bai et al., 2015), and/or a recently described population of TrkC fibers that innervate the vasculature (Morelli et al., 2021).

### Collateral sprouting of tyrosine hydroxylase expressing fibers into denervated skin after peripheral nerve injury

**Figure 7.**
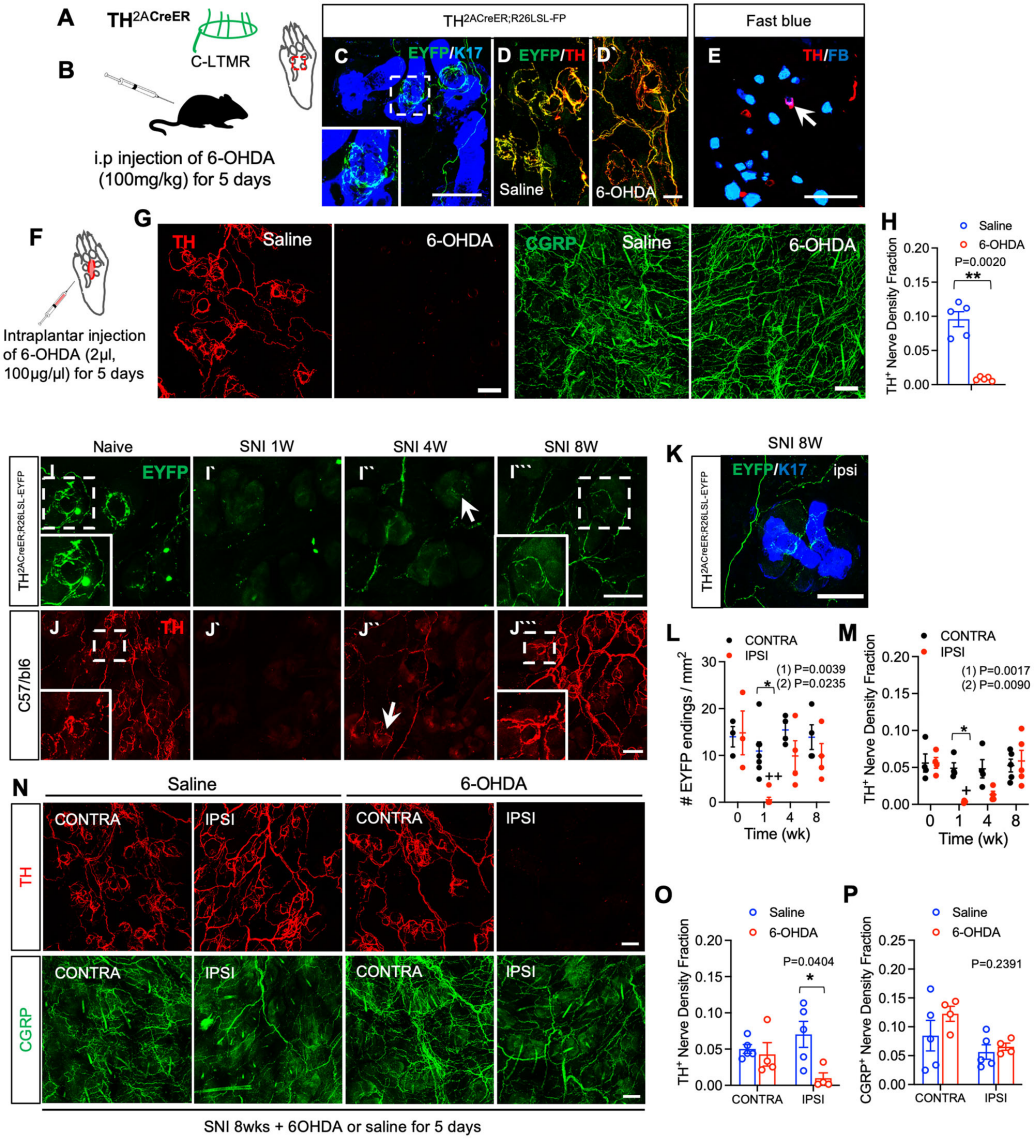
Sprouting of tyrosine hydroxylase expressing fibers into denervated skin after peripheral nerve injury. ***A***, Schematic of characteristic lanceolate endings of C-LTMRs labeled in TH^2ACreER^-Rosa26^LSL-EYFP^ mice. Modified from Abraira and Ginty (2013). ***B***, Schematic of systemic treatment with 6-OHDA to ablate sympathetic neurons. ***C***, Whole-mount immunostaining for EYFP (green) and K17 (blue) in hindpaw plantar hairy skin from TH^2ACreER^-Rosa26^LSL-EYFP^ mice. Inset, expanded view of EYFP^+^ lanceolate nerve ending. ***D***, Whole mount immunostaining for EYFP (green) and TH (red) in the hindpaw plantar hairy skin of naïve TH^2ACreER^-Rosa26^LSL-EYFP^ mice after i.p. injection of saline (panel ***D***) and 6-OHDA (panel ***D`***) for 5 consecutive days. ***E***, Immunostaining for TH (red) with FB retrogradely labeled plantar hairy skin-innervating neurons (blue) in lumbar DRGs from TH^2ACreER^-Rosa26^LSL-EYFP^ mice. Arrow indicates retrogradely labeled neuron. Scale bar, 100 μm. ***F***, Schematic for intraplantar injections of 6-OHDA (2 μl, 100 μg/μl in saline) for 5 consecutive days. ***G***, Whole mount immunostaining for TH (red) and CGRP (green) in the hindpaw plantar hairy skin after local intraplantar injection of saline (left panel) or 6-OHDA (right panel) into C57BL6 mice. ***H***, Quantification of TH nerve density fraction in experiment shown in panel (***B***) (saline, blue, *n* = 5; 6-OHDA, red, *n* = 5). ***I***, In TH^2ACreER^-Rosa26^LSL-EYFP^ mouse, whole mount immunostaining for tdTomato (red) in the ipsilateral hindpaw plantar hairy skin before and 1, 4, and 8 weeks after SNI. Inset, expanded view of GFP^+^ nerve ending. Scale bar, 100 μm. ***J***, Whole-mount immunostaining for TH (red) in the ipsilateral hindpaw plantar hairy skin of C57BL6 mouse before and 1, 4, and 8 weeks after SNI. ***K***, Whole-mount immunostaining for EYFP^+^ nerve fibers (green) in TH^2ACreER^-Rosa26^LSL-EYFP^ 8 weeks after SNI, showing relationship of loose circumferential endings to K17^+^ hair follicles (blue) hair follicles. ***L***, Quantification of EYFP^+^ nerve ending density in contralateral (black) and ipsilateral (red) hindpaw plantar hairy skin at the indicated times before and after SNI (*n* = 3–7) in TH^2ACreER^-Rosa26^LSL-EYFP^ mice. ***M***, Quantification of TH^+^ nerve density fraction in contralateral (black) and ipsilateral (red) hindpaw plantar hairy at the indicated times before and after SNI (*n* = 4–5) in C57Bl6 mice. ***N***, Whole mount immunostaining for TH (red) and CGRP (green), in the contralateral and ipsilateral hindpaw plantar hairy skin of C57BL6 mice 8 weeks after SNI and following i.p. injection of saline or 6-OHDA for 5 consecutive days as in panel (***B***). ***O***, Quantification of TH^+^ nerve density fraction in hindpaw hairy plantar skin in the experiment shown in panel (***N***) (saline, blue, *n* = 4; 6-OHDA, red, *n* = 5). ***P***, Quantification of CGRP^+^ nerve density fractions in the experiment shown in panel (***N***) (saline, blue, *n* = 5; 6-OHDA, red, *n* = 4). Data are presented as mean ± SEM. In panel ***H***, paired two-tailed Student's *t* test. **p = 0.0020, saline versus 6-OHDA. In panels ***L*** and ***M***, (1) indicates overall *p*-value for difference between baseline and ipsilateral hindpaw s over time using one-way ANOVA. Results of Bonferroni post hoc correction shown as: ^+^*p* < 0.05; ^++^*p* < 0.01. (2) Indicates overall *p*-value for difference between ipsilateral and contralateral paws over time using two-way ANOVA. Results of Bonferroni post hoc correction shown as: *p < 0.05. In panels ***O*** and ***P***, overall *p*-value for difference between saline and 6-OHDA treated mice using two-way ANOVA. Results of Bonferroni post hoc correction shown as: *p < 0.05. Saline versus 6OHDA. Full statistical details can be found in Table 1.

Next, we explored whether plantar hairy skin also contains nonmyelinated C-LTMRs, which form lanceolate endings around hair follicles, and can be genetically labeled in tyrosine hydroxylase (TH)^2ACreER^-Rosa26^LSL-EYFP^ mice by postnatal 4-OHT treatment (Fig. 7*A*; Li et al., 2011). In naïve mice of this genotype, we observed nerve fibers positive for enhanced yellow fluorescent protein (EYFP) in plantar hairy skin with endings surrounding hair follicles that exhibited a lanceolate but somewhat disheveled appearance (Fig. 7*C,I*). Both the fibers and their endings colabeled extensively with anti-TH antibody (Fig. 7*D*). A similar pattern of TH labeling was observed in the hairy plantar skin of wild-type C57BL6 mice (Fig. 7*I*; Kim et al., 2014). Further evidence for projection of C-LTMRs into hairy plantar skin was obtained via local retrograde FB labeling of neurons innervating this territory in C57BL6 mice, as TH labeling of lumbar DRG neuronal cell bodies revealed a subset of FB/TH double-positive neurons (Fig. 7*E*). Together, these experiments indicated that at least a subset of the TH positive fibers in naïve hairy plantar skin are of DRG origin and, based on their lanceolate ending structure, are likely C-LTMRs. To examine whether some of the TH^+^ fibers might alternatively arise from sympathetic neurons, we performed intraperitoneal (i.p.) injection of 6-hydroxydopamine (6-OHDA, 100 mg/kg) for 5 consecutive days to chemically ablate the sympathetic neurons (Kostrzewa and Jacobowitz, 1974) while sparing C-LTMRs (Li et al., 2011). This treatment failed to eliminate either TH EYFP^+^ or anti-TH^+^ nerve fibers in plantar hairy skin, consistent with the presence of C-LTMRs (Fig. 7*D`*). Interestingly, local intraplantar injection of 6-OHDA eliminated nearly all TH positive nerve fibers (Fig. 7*F,G,H*). It is unclear if this represents a nonspecific neurotoxicity resulting from a locally administered, high dose of this reagent, as has been reported (Michel and Hefti, 1990), though we observed no overt loss of CGRP^+^ fibers with local 6-OHDA treatment (Fig. 7*G*).

We next performed SNI surgery on TH^2ACreER^-Rosa26^LSL-EYFP^ and C57BL6 mice to investigate whether intact TH-expressing neurons would sprout into denervated skin after peripheral nerve injury. As with other fiber types examined, we observed a significant reduction in TH EYFP^+^ nerve fiber density 7 d after SNI in TH^2ACreER^-Rosa26^LSL-EYFP^ mice. However, TH EYFP^+^ nerve fibers sprouted new collaterals into denervated skin by 4 and 8 weeks (Fig. 7*I–I```,L*). These fibers formed loose circumferential endings around hair follicles (Fig. 7*I```,K*) that appeared to lack the lanceolate processes characteristic of C-LTMR endings. In C57BL6 mice, we also saw a similar pattern, with significant reduction of TH^+^-IR nerve fibers 7 d after injury and innervation that began to return by 4 weeks and that largely recovered by 8 weeks (Fig. 7*J–J```,M*). To determine whether these sprouted fibers included C-LTMRs and/or sympathetic nerve fibers, we injected 6-OHDA intraperitoneally for 5 consecutive days at 8 weeks after SNI. In the ipsilateral plantar hairy skin of the 6-OHDA-treated mice, TH^+^ nerve fiber density was significantly reduced compared to that in the saline-treated mice (Fig. 7*N,O*). In contrast, there was no difference in the contralateral plantar hairy skin of the two groups. Systemic treatment with 6-OHDA also did not significantly affect the collateral sprouting of CGRP nerve fibers (Fig. 7*N,P*). Together, these results suggest that whereas many of the TH^+^ nerve fibers observed in control skin are C-LTMRs, TH^+^ fibers exhibiting collateral reinnervation of skin after peripheral nerve injury appear to consist predominantly of sympathetic neurons, without evidence of C-LTMR sprouting.

### Collateral sprouts after nerve injury are functional

**Figure 8.**
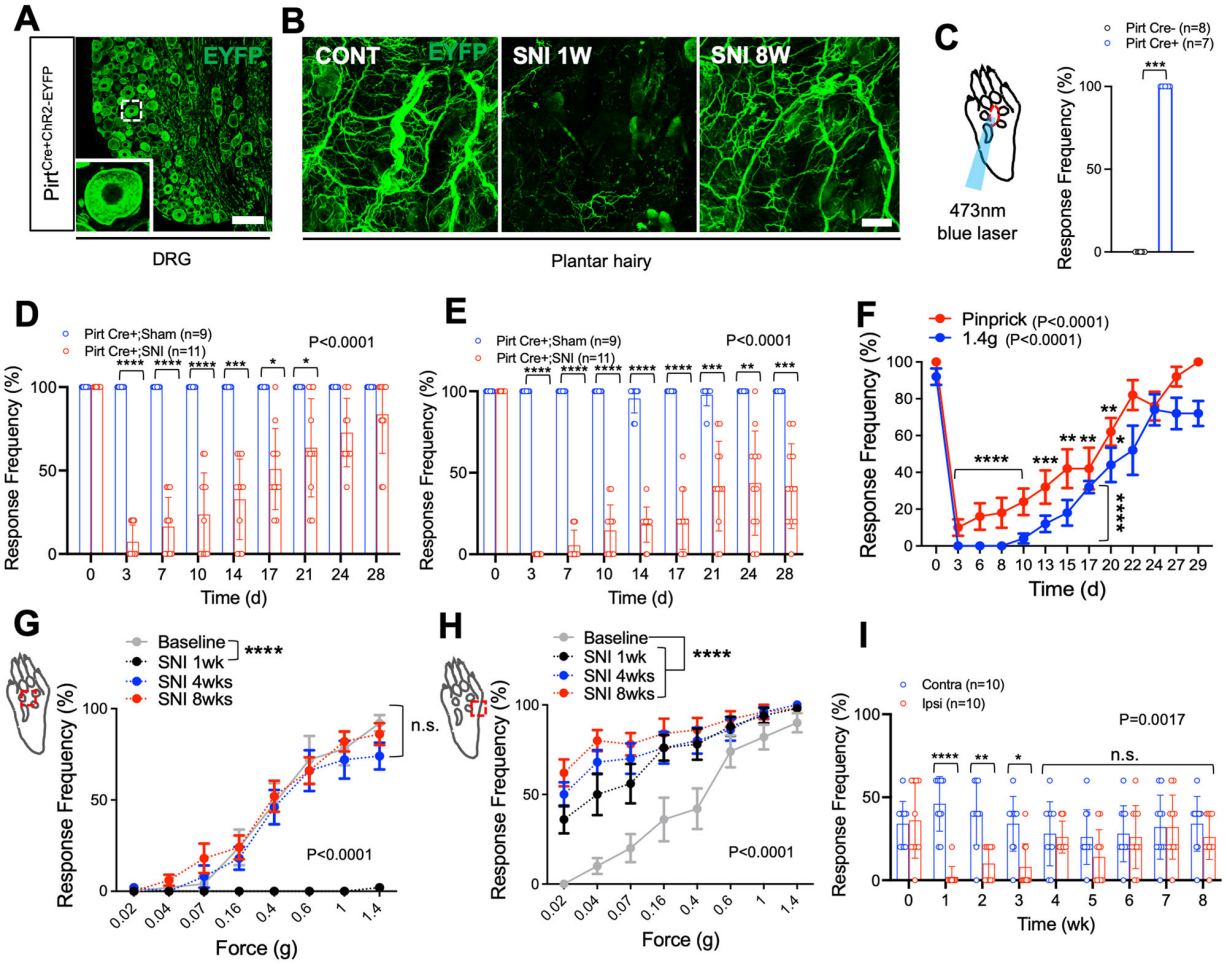
Collateral sprouting contributes to functional recovery of sensory neurons after peripheral nerve injury. ***A***, Immunostaining for EYFP (green) in lumbar DRGs from Pirt^Cre+^;Rosa26^LSL-ChR2-EYFP^ mice. Scale bar, 100 μm. ***B***, Whole mount immunostaining for EYFP (green) on the contralateral and ipsilateral hindpaw plantar hairy skin before and 1 and 8 weeks after SNI in Pirt^Cre+^;Rosa26^LSL-ChR2-EYFP^ mice. ***C***, Schematic of mouse paw stimulation using 473 nm blue laser (left) and the frequency of withdrawal from light stimulation of the hindpaw of Pirt^Cre+^;Rosa26^LSL-ChR2-EYFP^ and Pirt^Cre-^;Rosa26^LSL-ChR2-EYFP^ mice (right). Data are presented as mean ± SEM. Mann-Whitney test. ***p = 0.0002, Cre- versus Cre^+^. ***D***, Time course of 100 ms blue light-induced paw withdrawal frequency in the ipsilateral tibial nerve-stimulated plantar hair skin after SNI (*n* = 11) and sham (*n* = 9) surgery in Pirt^Cre+^;Rosa26^LSL-ChR2-EYFP^ mice. In panels ***D***, ***E***, and ***I***, circles represent data from individual mice. ***E***, Time course of 10 ms blue light-induced paw withdrawal frequency in the ipsilateral tibial nerve-stimulated plantar hair skin after SNI (*n* = 11) and sham (*n* = 9) surgery in Pirt^Cre+^;Rosa26^LSL-ChR2-EYFP^ mice. In panels ***D*** and ***E***, two-way repeated measures ANOVA with *p*-value from overall comparison between groups over time shown at top. Results of Bonferroni post hoc correction shown as: *p < 0.05; **p < 0.01; ***p < 0.001; ****p < 0.0001, sham versus SNI. All quantified data are presented as mean ± SEM. In panels ***C,F,G,H***, symbols represent individual mice. ***F***, Time course of pinprick (red) or 1.4 g von Frey filament (blue) evoked mechanical sensitivity, after SNI, in the ipsilateral tibial nerve-innervated plantar hairy skin of C57BL6 mice (*n* = 10). One-way repeated measures ANOVA with *p*-value for difference between baseline and ipsilateral hindpaw s over time shown at bottom. Results of Bonferroni post hoc correction shown as: *p < 0.05; **p < 0.01; ****p < 0.0001. ***G***, Punctate mechanical sensitivity measured across forces in the ipsilateral tibial nerve-innervated plantar hairy skin of C57BL6 mice at baseline (grey), and 1 (black), 4 (blue), and 8 weeks (red) after SNI (*n* = 10). Two-way repeated measures ANOVA with *p*-value from overall comparison between groups over time shown at bottom. Results of Bonferroni post hoc correction shown as: ****p < 0.0001, baseline versus SNI. ***H***, Punctate mechanical sensitivity measured across forces using von Frey filaments in the ipsilateral sural nerve-innervated lateral hindpaw plantar skin at baseline (grey), and 1 (black), 4 (blue), and 8 weeks (red) after SNI (*n* = 10). Two-way ANOVA with *p*-value from overall comparison between groups over time shown at bottom. Results of Bonferroni post hoc correction shown as: ****p < 0.0001, baseline versus SNI. ***I***, Time course of hindpaw sensitivity to 0.4 g von Frey filaments, after SNI, in the contralateral and ipsilateral tibial nerve-innervated plantar hairy skin of C57BL6 mice (*n* = 10). Two-way repeated measures ANOVA with *p*-value from overall comparison between groups over time shown at top. Results of Bonferroni post hoc correction shown as: *p < 0.05; **p < 0.01; ****p < 0.0001, contra versus ipsi. Full statistical details can be found in Table 1.

To investigate whether the fibers that sprout collaterally after nerve injury are functional, we used optogenetic stimulation of Pirt^Cre^;Rosa26^LSL-ChR2-EYFP^ mice, in which the light-gated ion channel channelrhodopsin-2 (ChR2), fused to EYFP is expressed in the vast majority of DRG neurons. Immunostaining for EYFP confirmed widespread expression not only in nearly all DRG neuronal cell bodies (Fig. 8*A*), but also in their terminals in plantar skin (Fig. 8*B*). A major reduction in ChR2-EYFP^+^ nerve fibers was observed in the hindpaw plantar hairy skin 1 week after SNI. However, intact ChR2-EYFP^+^ neurons sprouted collaterals into denervated skin by 8 weeks (Fig. 8*B*). We used a 473 nm laser to optogenetically stimulate these fibers. Light pulses (10 or 100 ms) delivered precisely to the hindpaw plantar hairy skin using an optical fiber cable evoked a strong paw withdrawal behavior in naïve Pirt^Cre^;Rosa26^LSL-ChR2-EYFP^ mice, but not controls lacking Cre (Fig. 8*C*). Response frequency to 100 ms blue light stimuli on the ipsilateral hindpaw of Pirt^Cre^;Rosa26^LSL-ChR2-EYFP^ mice dropped dramatically by 3 d after SNI, but returned gradually to basal level over 28 d (Fig. 8*D*). The response frequency to the 10 ms blue light stimulus on the ipsilateral hindpaw also dropped dramatically by day 3 after SNI and gradually recovered, but did not reach basal level by 28 d (Fig. 8*E*). These findings indicated substantial, but not complete, recovery of functional sensory afferents within this time frame.

Given the altered complement of sensory fibers in the plantar hairy skin after collateral sprouting, namely a predominance of nociceptors and a paucity of LTMRs, we sought to quantitatively examine responses to natural, tactile stimuli. We therefore measured behavioral responsiveness to punctate mechanical stimuli, applied to the plantar hairy skin of the hindpaw. Application of either a high force (1.4 g) von Frey filament or an Austerlitz pin (Latremoliere et al., 2018) evoked paw withdrawal with high efficiency in naïve C57BL6 mice. Three days after SNI surgery, the response frequencies to both stimuli, when delivered to the denervated hind-paw skin, decreased markedly. However, responsiveness returned gradually to basal levels by ∼4 weeks (Fig. 8*F*). Assay with von Frey filaments over a full range of forces confirmed that SNI produced a loss of touch-evoked responses at 1 week. Yet, touch sensitivity recovered to a pattern indistinguishable from baseline by 4 weeks, and exhibited a similar pattern at 8 weeks with no shift in the stimulus-response curve, suggesting there was a lack of mechanical hypersensitivity at either time (Fig. 8*G*). We further confirmed the absence of mechanical hypersensitivity throughout this time window by performing the von Frey assay once a week with a filament of intermediate force (0.4 g) applied to the plantar hairy skin (Fig. 8*I*). In contrast, the expected SNI-induced mechanical hypersensitivity was observed in the lateral plantar hindpaw skin (sural dermatome) at 1 week and persisted through 8 weeks (Fig. 8*H*). These results suggest that collateral sprouting contributes to functional recovery of sensory neurons in denervated skin, and that even behavioral responses to relatively weak mechanical stimuli can be restored, despite the apparently limited participation of LTMRs in collateral sprouting.

## Discussion

In this study, we assessed collateral sprouting following SNI surgery in the mouse. After initial denervation in tibial nerve skin territory, we observed reinnervation attributable to collateral sprouting of neighboring uninjured afferents. However, sprouting was not uniform among neuronal subtypes. Peptidergic nociceptors, both myelinated and unmyelinated, showed extensive sprouting into denervated skin. Unmyelinated nociceptors/pruriceptors showed a burst of sprouting by 4 weeks, but those terminals exhibited a trend towards regression by 8 weeks. Aβ RA-LTMRs and C-LTMRs, both of which innervated hairy plantar skin at baseline, failed to convincingly sprout, even 8 weeks after denervation. However, a subset of TrkC lineage neurons, including some that were myelinated and most likely Aβ field LTMRs exhibited occasional sprouting.

Prior studies in rat reported collateral sprouting by spared peptidergic and NF-H-positive sensory fibers (Duraku et al., 2012, 2013; Cobianchi et al., 2014; Nascimento et al., 2015). Another study used the mouse SNI model and serial in vivo microscopy to show that sural nerve nociceptive neurons sprouted collaterally into digit-tip glabrous skin in the tibial nerve territory (Gangadharan et al., 2022). In our study, as well as several performed in rat (Duraku et al., 2013; Cobianchi et al., 2014; Nascimento et al., 2015; Lakatos et al., 2020), sprouting peptidergic and nonpeptidergic neurons reinnervated not only the upper dermis, but also the epidermis. In contrast, Gangadharan et al. (2022) reported that sprouting nociceptive neurons remained at the dermal-epidermal boundary, without epidermal penetration, even 42 weeks postinjury. This difference may be related to the distinct anatomical site (i.e., digit tip) assayed in that study. Further evidence for nociceptor collateral sprouting in rat has come from functional assays (Doucette and Diamond, 1987; Lakatos et al., 2020). In the mouse SNI model, skin-nerve preparation electrophysiological recordings directly showed expansion of receptive fields of sural nociceptive C fibers into tibial skin territory (Gangadharan et al., 2022).

Our finding that some LTMRs show little or no collateral sprouting is generally in line with prior reports using behavioral, anatomical, or electrophysiological methods (Jackson and Diamond, 1984; Gangadharan et al., 2022). However, as noted above, in a subset of mice we did observe sprouting by TrkC lineage neurons that morphologically resembled Aβ-Field LTMRs. Consistent with this notion, in mice exhibiting more extensive TrkC lineage neuron sprouting, a larger percentage of sprouted TrkC neurons were NFH positive. Should this be confirmed electrophysiologically, it might suggest an inefficient LTMR sprouting process potentially amenable to augmentation, if therapeutically warranted. It is also possible that failure of NFH protein to enter sprouting fibers may lead to an underestimate of sprouting by myelinated neurons.

We also observed innervation of hairy plantar skin by TH^+^ nerve fibers before and 8 weeks after SNI surgery. In naïve mice, many of these were resistant to ablation by systemic 6-OHDA, consistent with C-LTMRs (Morelli et al., 2021). In contrast, 8 weeks after SNI surgery, sprouted TH^+^ afferents (but not TH^+^ fibers in the naïve contralateral paw) were largely eliminated by systemic 6-OHDA, consistent with a predominantly sympathetic neuron origin (Michel and Hefti, 1990). Prior studies have reported sympathetic collateral sprouting into denervated rat skin (Gloster and Diamond, 1995; Nascimento et al., 2015). In one such study (Nascimento et al., 2015), the sympathetic fibers were present only transiently (4–6 weeks), whereas in our present study, they persisted at 8 weeks. Gangadharan et al. (2022) observed cutaneous sprouting by TH^+^ fibers in a subset of denervated mice, but inferred a DRG origin. Sympathetic neurons also sprout into the DRG following nerve injury and contribute to spontaneous firing by clusters of neurons and to spontaneous pain behaviors (Zheng et al., 2022). Details of sympathetic neuron structure and function in naïve and reinnervated plantar skin therefore merit future attention. We also cannot exclude the presence of TH^+^ TrkC lineage sensory neurons that form endings around blood vessels rather than hair follicles (Morelli et al., 2021).

Differential abundance of neurotrophins in denervated skin might be one contributor to differential subtype sprouting. One feature shared by sympathetic and peptidergic neurons is responsiveness to nerve growth factor (NGF). Moreover, anti-NGF antibodies suppress collateral sprouting, but not conventional regeneration, by both neuronal populations (Diamond et al., 1987; Diamond and Foerster, 1992; Gloster and Diamond, 1995). However, the lack of NGF responsiveness of adult nonpeptidergic nociceptors, which also sprout, albeit with an apparently transient timecourse, suggests involvement of additional factors whose abundance might exhibit similarly transient kinetics. Other possible contributors to differential sprouting include differential intrinsic neuronal activity and/or mechanosensitivity (Gangadharan et al., 2022), differential intrinsic expression of sprouting associated genes (Harrison et al., 2015; Lemaitre et al., 2020; Gangadharan et al., 2022) or differential intrinsic expression of receptors for factors released during Wallerian degeneration, which may promote sprouting (Collyer et al., 2014). We also cannot exclude the possibility that the apparent difference in sprouting propensity between Aβ-RA-LTMRs and TrkC lineage fibers reflects the greater baseline cutaneous density of the latter.

In parallel with a sequence of denervation, then gradual reinnervation, we observed a loss and then gradual recovery of behavioral responses to mechanical stimulation in denervated hairy plantar skin that reached baseline levels by 4 weeks. Responses to optogenetic activation of sensory neurons were also lost after injury, but partially recovered towards baseline levels within 4 weeks. These data confirm the functionality of collaterally sprouted fibers. Lacking a system that would permit us to focus heat specifically on hairy plantar skin, we could not monitor thermosensory recovery. Our functional studies revealed two interesting findings. First, we saw full recovery of von Frey force-response relationship, including at low forces, despite an incomplete repertoire of LTMR sprouting. Prior data have implicated MrgprD^+^ nonpeptidergic nociceptors/pruriceptors, but not peptidergic C fibers, in the response to punctate mechanical stimuli in naïve mice (Cavanaugh et al., 2009). Yet, significant von Frey responses remain even after nociceptor ablation (Abrahamsen et al., 2008). LTMRs, on the other hand, have been proposed to qualitatively shape and in some cases attenuate these responses (Arcourt et al., 2017). More sophisticated behavioral assays, including high-speed video analysis (Arcourt et al., 2017; Abdus-Saboor et al., 2019) will be required to determine whether the sprouted nociceptors recapitulate the full range of pre-injury mechanical responsiveness. Such assays should also be correlated with TrkC lineage labeling, to determine whether inconsistent sprouting by these fibers influences behavioral responses. Second, despite the gradual recovery of normative punctate nociception, at no time during the 8 weeks course of our experiments did we observe mechanical hypersensitivity in the reinnervated area. In the rat sciatic nerve transection model, Cobianchi et al. (2014) reported loss of sensory function in denervated skin, followed by recovery, with a brief, transient period of mechanical hypersensitivity ∼50 d after sciatic transection. A prior study of humans undergoing C7 spinal nerve transposition surgery reported transient mechanical and cold hypersensitivity in denervated skin in a subset of patients that was likely attributable to collateral sprouting (Ali et al., 2002). Extensive analysis of sensory recovery from denervation following SNI in the mouse was performed by Gangadharan et al. (2022). Within the time window common between our two studies, our respective findings are similar, namely full and faithful recovery of von Frey force-response characteristics in the tibial territory by 8 weeks. Remarkably, however, while in their study mechanosensitivity remained relatively stable for the ensuing 8 weeks, 16 weeks after SNI the mice began to develop mechanical hypersensitivity in the tibial territory that was paralleled by supranormal sensitivity of C fibers innervating tibial skin. Concomitant with this hypersensitivity, the authors observed de novo innervation of Meissner's corpuscles in tibial territory digit tip skin by nociceptive fibers, providing one possible explanation for their mechanical hypersensitivity. We do not know if hyperalgesia would also have been seen in our mice at times beyond 8 weeks. It is also not clear to what extent glabrous versus hairy plantar skin was stimulated during the functional studies of Gangadharan et al., though their skin-nerve recordings appear to have focused predominantly on glabrous skin. It is also possible that the anatomical differences between our two studies, such as presence vs lack of epidermal reinnervation, sprouting by TrkC lineage neurons, or prevalence of sympathetic sprouting would have functional consequences at later time points.

In summary, we provide evidence for subtype-specific collateral reinnervation of hairy plantar skin in the mouse SNI model that is accompanied by robust recovery of punctate mechanosensitivity within 4 weeks, despite a paucity of LTMRs. Recovery of tactile sensation was seen without hypersensitivity, despite the fact that a portion of the sprouting originated from the adjacent sural dermatome, which demonstrated characteristic long-lasting hypersensitivity. Collectively, our results highlight the importance of future studies to determine what drives subtype-specific sprouting and how neuronal plasticity varies across the receptive fields of reinnervated and spared skin territories, as well as other tissues such as muscle. They also have implications for strategies to enhance sensory recovery and limit neuropathic pain after peripheral nerve injury.
